# From photosynthetic electron flow to gene regulation: redox signal transduction in cyanobacteria

**DOI:** 10.1093/femsre/fuag030

**Published:** 2026-07-15

**Authors:** Zachary Johnson, Bin Yang, Pavlo Bohutskyi

**Affiliations:** Biological Sciences Division, Pacific Northwest National Laboratory, Richland, WA 99352, USA; Bioproducts, Sciences, and Engineering Laboratory (BSEL), Department of Biological Systems Engineering, Washington State University, Richland, WA 99354, USA; Bioproducts, Sciences, and Engineering Laboratory (BSEL), Department of Biological Systems Engineering, Washington State University, Richland, WA 99354, USA; Biological Sciences Division, Pacific Northwest National Laboratory, Richland, WA 99352, USA; Department of Biological Systems Engineering, Washington State University, Pullman, WA 99164, USA

**Keywords:** redox homeostasis, transcriptional regulation, metalloregulators, reactive oxygen species, thiol switches, iron-sulfur clusters

## Abstract

In cyanobacteria, the free-living ancestors of chloroplasts, photosynthesis simultaneously sustains growth and generates reactive oxygen species (ROS) that damage proteins, lipids, and DNA when light capture outpaces carbon fixation. Maintaining redox balance, therefore, requires cells to read photosynthetic electron flow as a signal that continuously tunes gene expression and protein activity. This review traces how these redox signals are transduced to transcription machinery through three routes: membrane-localized sensors, cytoplasmic redox sensors downstream of photosystem I, and ROS generated when electron sinks are saturated. Membrane-bound histidine kinases (two-component systems) relay the redox state of the plastoquinone pool to control photosystem remodeling, pigment biosynthesis, and circadian timing. Cytoplasmic one-component regulators, by contrast, sense redox directly through thiol-disulfide switches, glutathionylation, iron-sulfur clusters, and metal-catalyzed oxidation to control photosystem-cofactor, electron-carrier, and transition-metal homeostasis. Because many of these regulators persist in algal and plant chloroplasts, cyanobacteria illuminate principles of redox control across photosynthetic eukaryotes. Post-transcriptional and translational control further shapes redox-dependent gene expression programs through transcript stability, ribosome assembly, and translation initiation, extending redox regulation beyond transcription to every step of protein synthesis and even activity modulation. Finally, we connect redox regulation to photosynthetic physiology, stress resilience, and the rational engineering of cyanobacteria for sustainable bioproduction.

## Teaser

Integrating recent structural, post-translational, and systems-level advances, this review traces how photosynthetic electron flow acts as a signal controlling gene expression in cyanobacteria, mediated by membrane sensors of the plastoquinone pool and cytoplasmic redox sensors downstream of photosystem I, and by reactive oxygen species, and highlights these regulators as targets for engineering cyanobacteria for sustainable bioproduction.

## Introduction

Oxygenic photosynthesis evolved in cyanobacteria ∼3 billion years ago (∼3 Ga), leading to the accumulation of oxygen in Earth’s atmosphere during the Great Oxidation Event (∼2.4 Ga) (Sánchez-Baracaldo and Cardona 2020). As a strong electron acceptor, oxygen readily undergoes partial reduction, producing reactive oxygen species (ROS) that can damage biological molecules (Imlay 2003). At the same time, the emergence of oxygen enabled more efficient redox chemistry and greater ATP yield through oxidative phosphorylation (Davies 1995, Catling et al. 2005). These opposing selective pressures drove the evolution of regulatory systems that maintain cellular redox balance across all domains of life.

Cellular redox state reflects the balance between electron supply and demand within a cell. This balance is defined by the ratio of reduced to oxidized cofactors [NAD(H), NADP(H)] and, in cyanobacteria, by electron carriers such as ferredoxin. Thioredoxin and glutaredoxin transmit redox signals to target proteins via thiol-disulfide exchange, linking the redox state of these pools to protein activity (Mondal and Singh 2022). Cellular redox state also functions as a signal for resource allocation in response to environmental variability. In cyanobacteria, light availability, temperature, and nutrient supply collectively determine electron flow through metabolic networks, and changes in redox state provide a rapid, reversible signal that couples external conditions to intracellular physiology (Sharma et al. 2024).

Cyanobacteria are highly dependent on sensing the redox state of photosynthetic electron transport because it simultaneously serves as the primary source of metabolic reducing power and ROS (Hamilton 2019). Fluctuations in light intensity over the diurnal cycle directly modulate electron flow through the photosynthetic electron transport chain, producing predictable oscillations in oxygen evolution, ROS formation, and intracellular redox pools (Venkiteswaran et al. 2007). These cyclical patterns are linked to the evolution of the circadian clock (Edgar et al. 2012), where the redox state of the plastoquinone pool serves as an input to the KaiABC circadian oscillator, calibrating clock phase with photosynthetic activity (Edgar et al. 2012, Diamond et al. 2017). Clock-regulated mechanisms, in turn, control the intracellular redox state (Diamond et al. 2017, Tanaka et al. 2020), such that redox poise is both an input to and an output of the circadian system (McFarlane et al. 2019, Selim et al. 2023, Doello et al. 2025). Redox regulation consequently links photosynthetic metabolism, transcriptional control, and temporal coordination in cyanobacteria.

These same redox dynamics that govern diurnal physiology in laboratory strains operate at the global scale in natural populations and constrain performance in engineered systems. Marine picocyanobacteria such as *Prochlorococcus* and *Synechococcus* account for approximately one quarter of global oceanic primary production (Flombaum et al. 2013), making their redox physiology a determinant of carbon cycling, nutrient flux, and ecosystem productivity (Bagby and Chisholm 2015). In both natural and engineered contexts, oxidative stress and redox imbalance constrain photosynthetic efficiency, growth, and metabolic output (Latifi et al. 2009).

In bioengineering applications, redox state governs the distribution of metabolic flux among biomass formation, protective molecules, and storage compounds, and must be explicitly considered when optimizing bioproduction (Ducat et al. 2012, Kugler and Stensjö 2023). Engineered overproduction of free fatty acids generates reactive oxygen species and imposes oxidative stress on the host (Ruffing 2013), while high-light and photooxidative conditions upregulate carotenoid biosynthesis as part of the native antioxidant response (Latifi et al. 2009). Redox-dependent regulation controls the accumulation of glycogen and polyhydroxybutyrate (PHB) (Thiel et al. 2019, Lucius and Hagemann 2024). Storage molecules and sucrose have been engineered as electron sinks to improve photosynthetic efficiency (Santos-Merino et al. 2023, Muth-Pawlak et al. 2024), and redirecting electron flux through hydrogenase complexes has improved hydrogen productivity (Tiwari and Pandey 2012). Engineering synthetic control over redox-dependent pathways to minimize oxidative stress and rebalance electron flux, therefore, represents a promising strategy for improving photosynthetic productivity.

**Figure 1:**
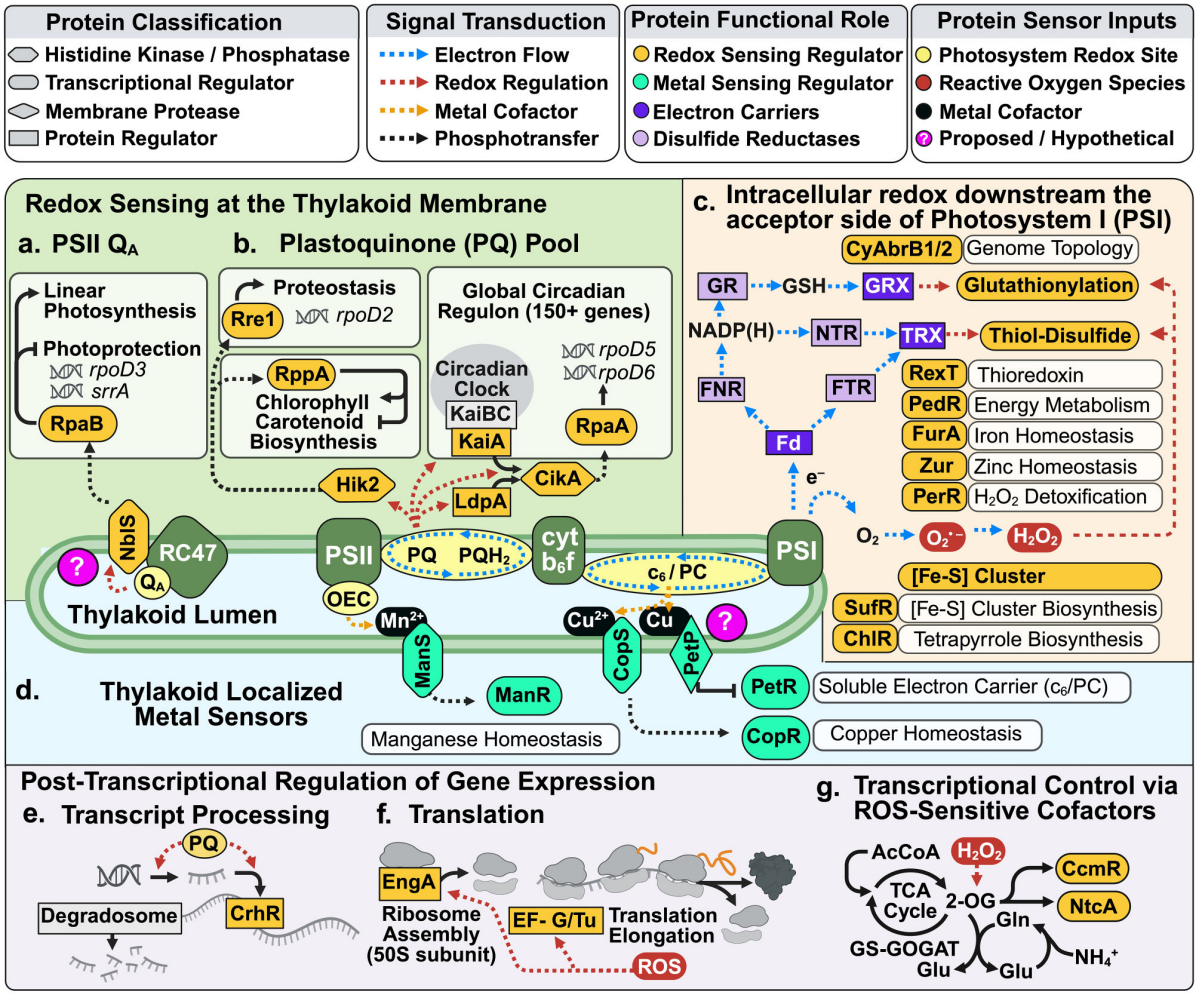
Redox signal transduction from the photosynthetic electron transport chain to transcriptional regulators in cyanobacteria. Redox signals originating from the photosynthetic electron transport chain (center) are transmitted to transcriptional regulators through three principal sensing sites: RC47-bound Q_A_, the plastoquinone (PQ) pool, and downstream of PSI. (a) Recent evidence suggests that NblS senses the photosystem II assembly intermediate RC47-bound Q_A_ redox state. NblS phosphorylates RpaB, thereby controlling a global photosynthesis regulon. (b) PQ pool redox state is sensed by Hik2 and by circadian clock proteins KaiA, CikA, and LdpA. Hik2 phosphorylates Rre1 (controlling a proteostasis regulon) and RppA (pigment biosynthesis). The clock integrates PQ redox inputs to control the activity of global response regulator RpaA, which in turn regulates the expression of over 150 genes. (c) On the PSI acceptor side, electrons are transferred to ferredoxin (Fd), distributing reducing equivalents through ferredoxin-NADP^+^ reductase (FNR) to NADP(H), and through ferredoxin-thioredoxin reductase (FTR) to the thioredoxin pool; NADPH in turn supplies glutathione reductase (GR) to maintain the GSH pool. When Fd becomes over-reduced, PSI reduces O_2_ to superoxide through the Mehler reaction, and superoxide is then dismutated to H_2_O_2_ by superoxide dismutase. The NADP(H) and GSH pools supply the disulfide-reducing proteins thioredoxin (TRX) and glutaredoxin (GRX), which modulate protein activity through thiol chemistry. One-component transcription factors downstream of PSI sense redox state via thiol-disulfide switches, glutathionylation, or ROS-/oxygen-sensitive iron-sulfur clusters. (d) Thylakoid-localized metal sensors ManS (Mn^2+^) and CopS (Cu^2+^) link luminal cofactor availability to transcriptional output. PetRP additionally links copper availability to the *petE*/*petJ* electron-carrier switch. PetP is hypothesized to be localized in the thylakoid membrane. (e) At the post-transcriptional level, the RNA helicase CrhR remodels transcript structure, determining if a transcript is translated or degraded. CrhR abundance is dependent on the PQ pool redox state. (f) Translation is further inhibited by ROS oxidizing cysteines in ribosome assembly factor EngA and translation elongation factors EF-G and EF-Tu. (g) ROS can further modulate metabolism to induce transcriptional changes: H_2_O_2_-driven depletion of 2-oxoglutarate (2-OG) controls the activity of NtcA (nitrogen metabolism) and CcmR (carbon metabolism). Created with BioRender.com.

Previous work has extensively characterized post-translational redox regulation of thiol-based switches in metabolic enzymes, allosteric regulatory complexes, and transcriptional regulators (McFarlane et al. 2019, Selim et al. 2023, Lucius and Hagemann 2024, Mantovani et al. 2024, Johnson et al. 2025a, Doello et al. 2025, Nikkanen et al. 2025, Sarkar et al. 2025, Kim et al. 2026). This review focuses on how photosynthetic electron flow drives redox signaling that regulates gene expression in cyanobacteria (Fig. 1), from transcriptional initiation (Fig. 1a–d) to post-transcriptional processing and translation (Fig. 1e–g). Membrane-localized sensors detect the balance between light energy input and downstream electron consumption at PSII and in the plastoquinone pool. These sensors provide redox inputs that calibrate photosystem expression (Fig. 1a) and circadian transcriptional programs (Fig. 1b). Thylakoid-localized metal sensors couple transition-metal availability to photosystem cofactor demand (Fig. 1d). Cytoplasmic one-component regulators sense the redox state of thioredoxin, glutaredoxin, and iron-sulfur pools maintained by PSI-derived electron flow through ferredoxin (Fig. 1c). Finally, we discuss the implications of redox regulation in bioproduction.

## Photosystem electron flow and redox signal transduction in cyanobacteria

To monitor photosynthetic electron flow, cyanobacteria sense the redox status of thylakoid quinones, the availability of photosystem cofactors in the thylakoid lumen, and the state of cytosolic redox pools. During linear electron transport, light energy captured by phycobilisomes (PBS) is transferred to both photosystem I (PSI) and photosystem II (PSII). At PSII this energy drives charge separation at P680, and electrons pass through pheophytin and the Q_A_/Q_B_ quinones to reduce the plastoquinone (PQ) pool to plastoquinol (PQH_2_), while the oxygen-evolving complex (OEC) replenishes P680^+^ by splitting water (Shen 2015). PQH_2_ is reoxidized at cytochrome b_6_f, which transfers electrons via copper-containing plastocyanin (PC) or iron-containing cytochrome c_6_ to PSI, where a second photochemical step reduces ferredoxin (Fd) (Grotjohann and Fromme 2005).

At the PSI acceptor side, Fd distributes electrons to ferredoxin-NADP^+^ reductase (FNR), which generates NADPH for the Calvin–Benson cycle, or to ferredoxin-thioredoxin reductase (FTR), which reduces thioredoxin (TRX) to relay redox signals to downstream protein targets. When these sinks become saturated, excess electrons are dissipated by flavodiiron proteins (Flv1–4) or transferred to O_2_, generating reactive oxygen species (ROS) (Fig. 1c) (Bersanini et al. 2014, Chaux et al. 2015). ROS broadly modify lipids, DNA, metabolites, and proteins (Imlay 2003). Protein thiol groups, iron-sulfur clusters, and metal-coordinating residues are particularly susceptible to oxidative modification and loss of activity (Stadtman 1990, Outten and Theil 2009, Wouters et al. 2010). TRX and glutaredoxin (GRX) reverse disulfide bond formation and glutathionylation at protein thiolates (Mondal and Singh 2022), while iron-sulfur cluster biogenesis restores the activity of Fe-S proteins damaged by cluster degradation (Pérard and Ollagnier de Choudens 2018).

Protein regulators exploit these same chemistries to transduce redox signals from distinct locations along the electron transport chain into transcriptional output. The PSII acceptor side (Q_A_) and PQ pool report the balance between light input and downstream electron consumption, thylakoid metal pools report cofactor availability for PSII and the soluble carriers, and the PSI acceptor side reports the status of cytoplasmic reducing power (Table 1).

**Table 1:** Redox-regulated gene expression regulators in cyanobacteria. Regulators are organized by sensing mechanism and relationship to photosynthetic electron flow. The Strain column indicates the model organism corresponding to the listed locus tags and in which the sensing mechanism was characterized; individual regulatory targets characterized in other strains are annotated parenthetically.

Regulator (locus)	Sensor (locus)	Regulator family	Strain	Redox-sensing mechanism	Regulatory targets	References
**PSII Q_A_ sensor:** *two-component system*						
RpaB ^**^†^**^ (1453)	NblS (0924)	OmpR	PCC 7942	NblS **PAS domain** senses RC47-bound Q_A_ redox state; bifunctional kinase/phosphatase	Photosynthesis regulon (HLR1 motif), *srrA, rpoD3, rpoD6, kaiBC*	Taylor and Zhulin 1999, Seki et al. 2007, Hanaoka and Tanaka 2008, López‐Redondo et al. 2010, Tsurumaki and Tanaka 2025
**Plastoquinone pool sensors:** *two-component system*						
Rre1 ^**^†^**^ (1860)	Hik2 (0453)	LuxR	PCC 7942	Hik2 **GAF domain [3Fe-4S] cluster** senses PQ redox; bifunctional kinase/phosphatase	Proteostasis (*groES, groEL1, groEL2, dnaK2, htpG*), *rpoD2/sigB*	Kobayashi et al. 2017, Bairagi et al. 2022, Hasegawa et al. 2024
RppA (sll0797)	Hik2 (slr1147)	OmpR	PCC 6803	Hik2 **GAF domain [3Fe-4S] cluster** senses PQ redox	Pigment biosynthesis (*crtP/pds, crtB, chlG, chlH*), *nrsBACD, rppAB*	Ibrahim et al. 2016, Bairagi et al. 2022, Yu et al. 2025
**Plastoquinone pool sensors:** *circadian clock protein regulator*						
RpaA ^**^†^**^ (0095)	KaiA (1218)	OmpR	PCC 7942	KaiA **PsR domain** binds oxidized quinones; aggregation prevents KaiC stimulation	Circadian global regulon (>150 genes), *opcA, zwf, gnd, hoxUYHW-hypAB*	Wood et al. 2010, Kim et al. 2012, Welkie et al. 2019
RpaA ^**^†^**^ (0095)	CikA (0644)	OmpR	PCC 7942	CikA **PsR domain** binds oxidized quinones; dissociation from KaiBC complex	Dephosphorylation of RpaA; gates circadian output	Schmitz et al. 2000, Ivleva et al. 2006, Gutu and O’Shea 2013, Welkie et al. 2019
RpaA ^**^†^**^ (0095)	LdpA (2416)	OmpR	PCC 7942	[**4Fe-4S] cluster** senses PQ redox state	Modulates CikA concentration; circadian phase control	Katayama et al. 2003, Ivleva et al. 2005, Welkie et al. 2019
**Metalated thiol switches:** *one-component system redox sensing downstream of PSI*						
Fur/FurA (all1691)	—	FUR	PCC 7120	**Active: C101** coordinates Fe^2+^/Mn^2+^; **C104**-**C133** intramolecular disulfide **Inactive: C101**-**C104** intramolecular disulfide; **C101**-**C101** intermolecular disulfide; **C141**-heme. **Reactivation:** TRX reduces **C141**-**C144**	Iron homeostasis (FUR box), pigment biosynthesis, oxidative stress response	Pellicer et al. 2012, Botello-Morte et al. 2016, Guío et al. 2021
Zur/FurB (all2473)	—	FUR	PCC 7120	**C93** redox state gates Zn^2+^ coordination at second regulatory site; possible heme coordination	Zinc homeostasis (*znuABC*), oxidative stress, *fur*	López-Gomollón et al. 2009, Sein-Echaluce et al. 2018, Kandari et al. 2021
PerR/FurC (alr0957)	—	FUR	PCC 7120	**H45/H98** ^**^††^**^: Fe^2+^-dependent MCO^**^a^**^ **C86** ^**^††^**^: intermolecular disulfide	H_2_O_2_ detoxification, carbon/nitrogen metabolism	Sarasa-Buisan et al. 2022a
**Non-metalated thiol switches:** *one-component system, redox sensing downstream of PSI*						
RexT (alr1867)	—	ArsR	PCC 7120	**C40**-**C41**: vicinal intramolecular disulfide	*trxA2*	Ehira and Ohmori 2012, Li et al. 2022
PedR (ssl0564)	—	LuxR	PCC 6803	**C80**: intermolecular disulfide; reduced by TrxM/TrxX	Chlorophyll-a biosynthesis (*chlL, chlN, chlB*), *slr1957, ndhD2, rpe, pedR-sll0296 operon*	Nakamura and Hihara 2006, Horiuchi et al. 2010
**Iron–sulfur cluster sensors:** *one-component system, redox sensing downstream of PSI*						
SufR (sll0088)	—	SufR	PCC 6803	**C164/C171/C206 [4Fe-4S]^2+^** coordination; cluster damage, iron limitation, or reduction derepresses targets	*sufBCDS* operon (Fe-S biosynthesis); *ftrC (PCC 7942)*	Wang et al. 2004, Shen et al. 2007
ChlR (sll1512)	—	MarR	PCC 6803	**C18/C25: [4Fe-4S]** coordination; O_2_-dependent cluster degradation inactivates DNA binding	*chlA2-ho2-hemN* (tetrapyrrole biosynthesis)	Aoki et al. 2012, Fujita et al. 2015
**Glutathionylation-glutaredoxin switches:** *one-component system, redox sensing downstream of PSI*						
CyAbrB2 (sll0822)	—	NAP	PCC 6803	**C34**: glutathionylation disrupts DNA binding	*hoxEFUYH, nifJ, atpT*, N/C metabolism, chromosome topology	Sakr et al. 2013, Song et al. 2022a, 2022b, Kariyazono and Osanai 2024
CyAbrB1 (sll0359)	—	NAP	PCC 6803	Glutathionylation, uncharacterized sensing residue	*hoxEFUYH, atpT, psbD2*, Chl-biosynthesis, carbohydrate metabolism	Sakr et al. 2013, Song et al. 2022a, 2022b,Yu et al. 2026
**Thylakoid-localized transition metal sensors:** *two-component system*						
ManR ^**^†^**^ (slr1837)	ManS ^**^b^**^ (slr0640)	OmpR	PCC 6803	ManS senses luminal Mn^2+^ (OEC cofactor); ManR **C154** ^**^††^**^ oxidized under PSII inhibition (DCMU) ^**^d^**^	*mntCAB* (Mn^2+^ ABC transporter)	Yamaguchi et al. 2002, Wang et al. 2022
CopR (sll0789)	CopS ^**^b^**^ (sll0790)	OmpR	PCC 6803	CopS senses luminal Cu^2+^ (plastocyanin cofactor); responds to photosystem disruption (DBMIB) ^**^e^**^	*copMRS, copBAC* (Cu^2+^ homeostasis)	Giner-Lamia et al. 2012, López-Maury et al. 2012
PetR (slr0240)	PetP ^**^b^**^ ^**^c^**^ (slr0241)	BlaI	PCC 6803	Copper activates PetP protease; irreversible PetR degradation	*petE* (plastocyanin), *petJ* (cytochrome c_6_)	García-Cañas et al. 2021

**Abbreviations:**  *GRX*, glutaredoxin; *NAP*, nucleoid-associated protein; *OEC*, oxygen-evolving complex; *PQ*, plastoquinone; *TRX*, thioredoxin; *MCO*, metal-catalyzed oxidation; *GSH*, glutathione (reduced); *GSSG*, glutathione disulfide (oxidized); *PSI*, photosystem I; *PSII*, photosystem II; *PsR*, pseudoreceiver domain.

**Locus tags:**  *Synechococcus elongatus* PCC 7942 (PCC 7942) identifiers are the four-digit suffix of Synpcc7942_XXXX.

*Synechocystis* sp. PCC 6803 (PCC 6803) and *Anabaena* sp. PCC 7120 (PCC 7120) use complete GenBank locus tags.

a MCO, metal-catalyzed oxidation: Fe^2+^-dependent Fenton reaction oxidizes coordinating histidine to 2-oxo-histidine.

b Transition metals function as photosystem cofactors; sensors are localized at the thylakoid membrane.

c PetP subcellular localization unresolved, predicted to be thylakoid membrane localized.

d DCMU, 3-(3,4-dichlorophenyl)-1,1-dimethylurea. PSII Q_B_ site inhibitor.

e DBMIB, 2,5-dibromo-6-isopropyl-3-methyl-1,4-benzoquinone. Cytochrome b_6_f Q_o_ site inhibitor, PQ pool reductant.

† Two-component system response regulator reduced by thioredoxin (PCC 6803, *in vitro*) (Kadowaki et al. 2015, Ibrahim et al. 2022).

†† Proposed residues based on high-throughput assay or structural modelling.

## Thylakoid membrane localized sensing of photosynthetic electron flow

Thylakoid-embedded two-component systems monitor photosynthetic electron flow through sensor histidine kinases whose input domains bind redox-active cofactors. Quinones, iron-sulfur clusters, reactive cysteines, and transition metals each serve as sensory inputs, with their oxidation or occupancy state gating kinase versus phosphatase activity. Signals are then transduced to cognate response regulators via phosphotransfer. Membrane localization places these sensors in direct contact with the quinone pool and thylakoid lumen, enabling readout of both PSII electron flux and the metal cofactor supply that limits photosystem assembly.

### NblS senses Q_A_ to globally remodel photosystem expression through RpaB

The NblS (Hik33)—RpaB TCS mediates global regulatory control over the photosynthetic electron transport chain by sensing changes in photosystem redox resulting from diverse environmental perturbations including photosynthetically active radiation (PAR) (Leusenko et al. 2024), ultraviolet radiation (UVR) (Chen et al. 2025), temperature (Fedurayev et al. 2018), and oxidative stress (Johnson et al. 2025c, Kanesaki et al. 2007, Fedurayev et al. 2018). Redox sensing is mediated by the histidine kinase NblS which is localized in the thylakoid membrane in *Synechococcus elongatus* PCC 7942 (PCC 7942), *Synechocystis* sp. PCC 6803 (PCC 6803), and *Synechococcus* sp. PCC 7002 (PCC 7002) (Dahlgren et al. 2021, Wang et al. 2022, Tsurumaki and Tanaka 2025), anchored by 2–3 transmembrane helices (Shimura et al. 2012, Leusenko et al. 2024). In PCC 7942, NblS has recently been proposed to sense Q_A_ by forming a complex with the PSII assembly intermediate [RC47-like-NblS_2_] (Tsurumaki and Tanaka 2025). While the exact redox-sensing mechanism remains uncharacterized (Tsurumaki and Tanaka 2025), the PAS domain (Taylor and Zhulin 1999) has been proposed to participate in redox sensing. PAS domains can control conformation and dimerization (Stuffle et al. 2021), which could switch NblS between autokinase and ADP-dependent phosphatase activity toward its cognate response regulator RpaB. NblS phosphorylation and dephosphorylation of RpaB has been demonstrated *in vitro* (López‐Redondo et al. 2010) and is consistent with the bifunctional architecture of its HisKA-HATPase_c domains. *In vivo*, NblS phosphatase activity is supported by RpaB phosphorylation levels dropping rapidly upon transition from low light (LL) to high light (HL) (Moronta-Barrios et al. 2012, Yasuda et al. 2020) and upon addition of electron transport inhibitors that reduce Q_A_ (Kato et al. 2022).

**Figure 2:**
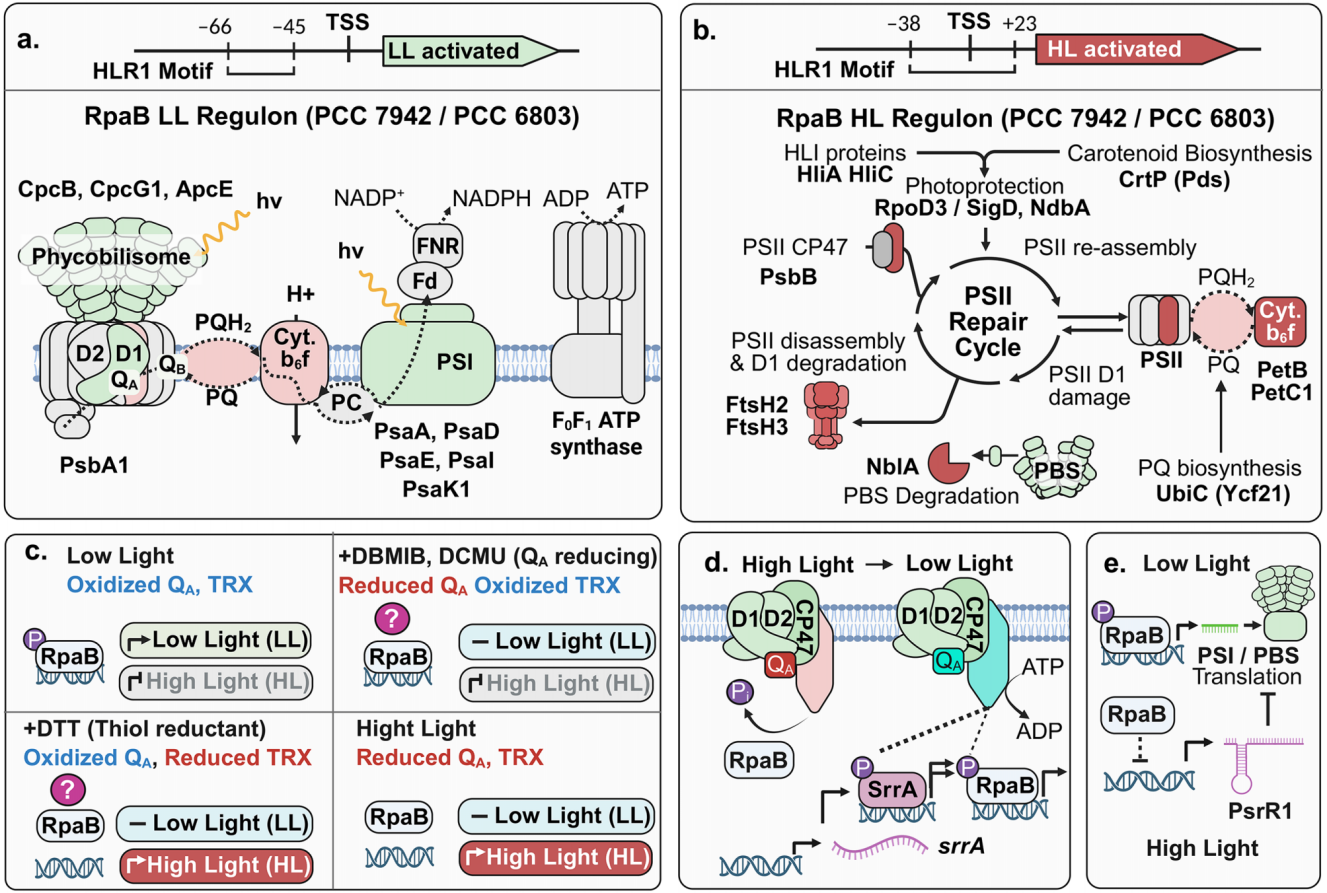
NblS RC47 Q_A_ sensing globally remodels photosynthesis through RpaB. (a) RpaB activates the low light (LL) regulon, binding the HLR1 motif –66 to –45 nt upstream of the transcription start site (TSS; PCC 6803). In PCC 7942 and PCC 6803, targets include low light activated PSII D1 isoform (*psbA1*), PSI genes (*psaA, psaD, psaE, psaI, psaK1*) and phycobilisome (PBS) genes (*cpcB, cpcG1, apcE*), and the F₀F₁ ATP synthase complex. (b) RpaB represses the high light (HL) regulon, binding an HLR1 motif that overlaps the TSS (–38 to +23 nt) to block RNA polymerase. The regulon is co-regulated by the HL-induced sigma factor *rpoD3*/*sigD*. Under HL (Q_A_ and TRX reduced) it is de-repressed, inducing photoprotection: PBS degradation (*nblA*), HL-inducible proteins (*hliA, hliC*), and carotenoid biosynthesis (*crtP*/*pds*), with HLI proteins and carotenoids contributing to chlorophyll photochemical quenching. Additional targets include PSII CP47 (*psbB*), cytochrome b_6_f (*petB, petC1*), type II NAD(P)H dehydrogenase (*ndbA*), the D1 protease (*ftsH2*/*ftsH3*), and PQ biosynthesis protein (*ubiC*/*ycf21*). (c) RpaB phosphorylation and DNA binding depend on the redox state of Q_A_ and PSI acceptor-side thiols (TRX). When both are oxidized, phosphorylated RpaB binds the HLR1 motif, activating the LL regulon and repressing the HL regulon. Reducing either Q_A_ or thiols dephosphorylates RpaB, but only thiol reduction (DTT) releases it from DNA, mimicking HL; the basis for this thiol-dependent release is unknown. Kato et al. 2022 propose that unphosphorylated RpaB still recruits RNA polymerase, whereas our model proposes the LL regulon is retained at basal transcription. (d) As PSII assembly is restored during the HL → LL transition, in PCC 7942, RpaB homolog SrrA (activated in the RpaB HL regulon) is preferentially phosphorylated over RpaB by NblS *in vitro*, potentially attenuating RpaB phosphorylation as conditions return to low stress. (e) In PCC 6803, RpaB and PsrR1 form a feedforward loop; on transition to high stress, PsrR1 blocks translation of existing PSI transcripts. Created with BioRender.com.

Phosphorylated RpaB functions as a global regulator of photosynthesis gene expression, recognizing an 18-bp consensus HLR1 DNA motif 5'-KTTACAWW-N_2_-KTTACAWW-3'. The same motif yields opposite outcomes depending on its position relative to the transcription start site (TSS) in PCC 7942 and PCC 6803: when HLR1 lies upstream (approximately −45 to −66 nt) RpaB activates transcription (Fig. 2a), whereas when it overlaps the TSS (–38 nt to +23), RpaB represses transcription (Fig. 2b) (Kappell et al. 2006, Riediger et al. 2018). Recent studies in PCC 6803 show RpaB DNA binding and phosphorylation are mechanistically separable, where RpaB activation requires both DNA binding and phosphorylation, while steric repression requires only occupancy and is largely independent of RpaB phosphorylation (Kato et al. 2022). Under low light, addition of the electron transport inhibitors DCMU and DBMIB, both of which reduce Q_A_, dephosphorylates RpaB but does not release it from binding the HLR1 motif, while addition of the reducing agent DTT, which mimics increased reducing equivalents at the PSI acceptor side, results in both dephosphorylation and loss of DNA binding. Direct redox sensing by the single conserved cysteine of RpaB (Cys59) was excluded by C59A substitution, leaving gaps for how reducing intracellular thiols results in RpaB dephosphorylation and loss of DNA binding. The net effect of this dual redox control is that bound RpaB can occupy three functionally distinct states in PCC 6803: phosphorylated and bound, dephosphorylated but bound, and released (Fig. 2c) (Kato et al. 2022). In PCC 7942, ChIP analysis has likewise shown that RpaB occupancy at target promoters is dynamic during high light stress (Hanaoka and Tanaka 2008).

In both organisms, RpaB is a positive regulator of low light PSII D1 isoform (*psbA1*), PSI, and PBS expression (Fig. 2a) while repressing transcription of sigma factor (*rpoD3*/*sigD*), PSII CP47 (*psbB*), cytochrome b_6_f, photoprotection and photosystem repair proteins (Fig. 2b) (Hanaoka and Tanaka 2008, Piechura et al. 2017, Riediger et al. 2019) . The RpaB regulon, however, is only partially conserved in PCC 7942 and PCC 6803, sharing 29 regulatory targets while ∼60% of computationally predicted binding sites in PCC 6803 have no homolog in PCC 7942 (Piechura et al. 2017, Riediger et al. 2019). Whether the three-state response characterized in PCC 6803 extends to PCC 7942 remains unclear, but distinct differences in RpaB regulatory feedback have been described between the two species. In PCC 7942, RpaB regulates type II sigma factors (*rpoD5/sigC, rpoD6*) and type III sigma factors (*sigF1, sigF2*) (Piechura et al. 2017, Johnson et al. 2025c), and specifically represses the type II sigma factor *rpoD3*/*sigD* (Seki et al. 2007) and the RpaB homolog *srrA* (López-Redondo et al. 2010). SrrA in turn competes with RpaB for phosphorylation by NblS in vitro, with SrrA being preferentially phosphorylated (López‐Redondo et al. 2010, Kato et al. 2011). Together, these findings suggest that SrrA may attenuate the RpaB-controlled response (Fig. 2d). In PCC 6803, regulation of *rpoD3/sigD* is conserved (Riediger et al. 2019), but no SrrA homolog exists (López‐Redondo et al. 2010). Instead, the RpaB regulon includes the SOS response regulator *lexA* (Riediger et al. 2019), and the trans-acting sRNA PsrR1, which base-pairs with PSI and PBS transcripts to block their translation under high light (Fig. 2e) (Kadowaki et al. 2016). In PCC 6803, PsrR1 functions as a feedforward loop, aiding RpaB-mediated transcriptional repression of PSI and PBS, while in PCC 7942, SrrA competes with RpaB for NblS phosphorylation, creating a feedback loop that buffers the high-light to low-light transition state.

### Hik2 PQ redox sensing coordinates pigment biosynthesis and proteostasis

**Figure 3:**
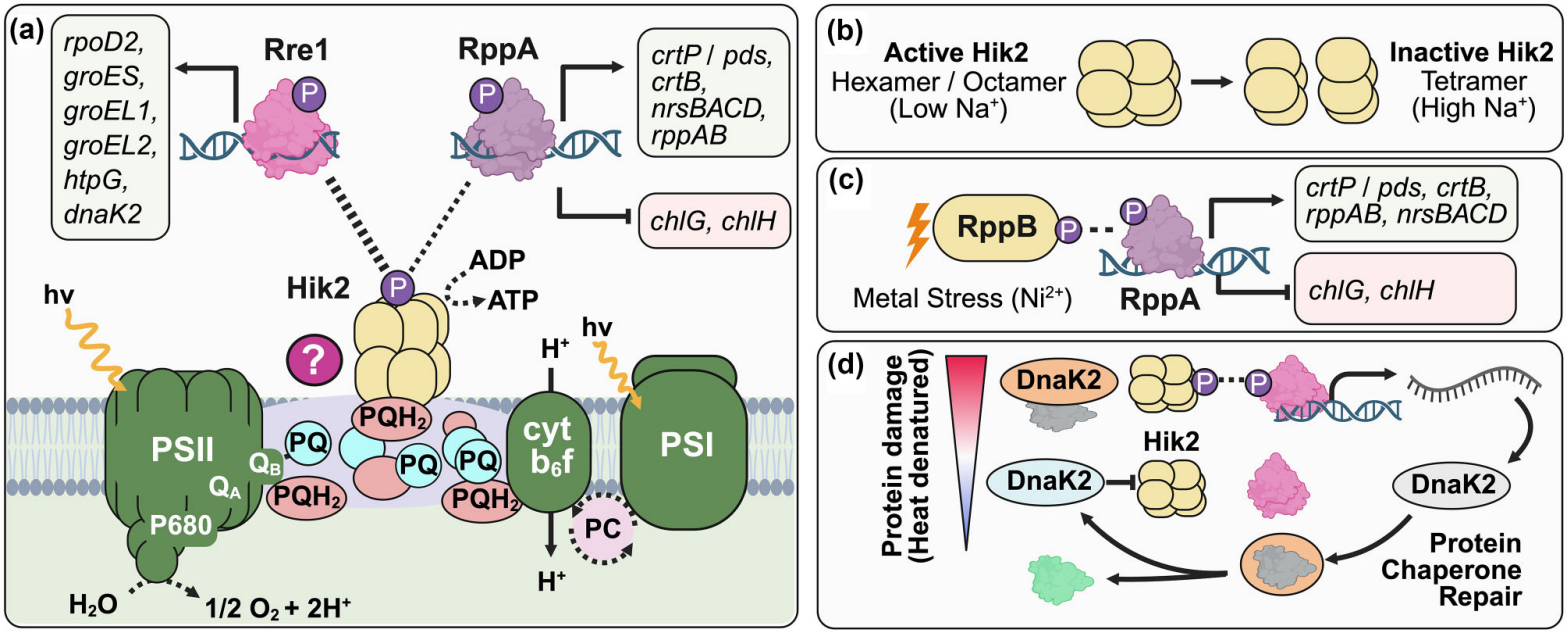
Hik2 PQ sensing regulates pigment biosynthesis (RppA) and proteostasis (Rre1). (a) Hik2 senses PQ pool redox state through its [3Fe-4S] cluster, though the activating redox direction remains unresolved. Hik2 preferentially phosphorylates Rre1, which activates the expression of protein chaperones and the type II sigma factor *rpoD2*/*sigB*. Hik2 simultaneously phosphorylates RppA, which activates the expression of carotenoid biosynthesis genes (*crtP/pds, crtB*), the nickel ABC transporter (*nrsBACD*), and its own operon (*rppAB*) while repressing chlorophyll biosynthesis (*chlG, chlH*). (b) Salt-dependent repression of Hik2 is controlled by protein oligomer state. Under low Na^+^ conditions, Hik2 forms active higher-order oligomers (hexamer/octamer); under high Na^+^ concentrations, it forms inactive tetramers (PCC 6803). (c) RppA plays a secondary role in response to heavy metals (*e.g*. Ni^2+^); this signal is transduced by the histidine kinase RppB (PCC 6803). (d) The DnaK2 transcriptional attenuation mechanism regulates Hik2 phosphorylation activity: low cellular protein damage represses Hik2 phosphorylation of Rre1, whereas high cellular protein damage titrates unbound DnaK2, preventing Hik2 repression (PCC 7942). Created with BioRender.com.

Hik2 is a highly conserved histidine kinase that lacks integral membrane domains and couples photosynthetic electron transport to transcriptional regulation in cyanobacteria and in plant chloroplasts, where the ortholog is the chloroplast sensor kinase (CSK) (Ibrahim et al. 2020). By binding [3Fe-4S] at the protein’s GAF domain, Hik2 directly senses the redox state of the plastoquinone (PQ) pool in cyanobacteria PCC 6803 and PCC 7942 (Fig. 3a) (Ibrahim et al. 2020, Bairagi et al. 2022). Hik2 also integrates a second signal: its oligomeric state is Na^+^-dependent in PCC 6803, where sodium ions inhibit autophosphorylation by converting higher-order oligomers to an inactive tetrameric form (Fig. 3b) (Ibrahim et al. 2016, Ibrahim et al. 2017).

The direction of Hik2 redox regulation remains unresolved. *In vitro* studies using PCC 6803 Hik2 show that reduced plastoquinone suppresses Hik2 autokinase activity, suggesting activation under PQ-oxidizing conditions (Ibrahim et al. 2020). However, *in vivo* Rre1 phosphorylation in PCC 7942 is enhanced under PQ-reducing conditions and blocked by exogenous quinone oxidation (Bairagi et al. 2022). Several factors may contribute to this discrepancy. First, the hypothesized bifunctional kinase/phosphatase activity of Hik2 (Ibrahim et al. 2016) may decouple *in vitro* autokinase activity (Ibrahim et al. 2020) from *in vivo* phospho-Rre1 measurements (Bairagi et al. 2022). Second, *in vivo* modulators absent from the reconstituted system, including Hik34, DnaK2, and Na^+^-dependent oligomeric regulation, may shift the net signaling output (Ibrahim et al. 2017, Hasegawa et al. 2024). Third, differences between recombinant and native protein [3Fe-4S] cluster occupancy and redox potential could alter the response threshold (Bairagi et al. 2022, Hasegawa et al. 2024).

RppA is an OmpR family response regulator identified in PCC 6803 with no known homolog in PCC 7942. RppA functions as both a transcriptional repressor and activator, recognizing the DNA binding motif 5'-AAMAWCTCYYWCCCC-3' (Yu et al. 2025). In its phosphorylated state, RppA simultaneously represses chlorophyll biosynthesis (*chlG, chlH*) and activates carotenoid biosynthesis (*crtP/pds, crtB*) (Yu et al. 2025). This dual output shifts cofactor flux from light harvesting to photoprotection, consistent with a role in mitigating photodamage when PQ pool reduction indicates excess excitation pressure (Shalygo et al. 2009). Phosphorylated by the histidine kinase RppB under heavy metal stress, RppA also activates its own operon (*rppAB*) and the nickel tolerance operon (*nrsBACD*) under heavy metal stress (Fig. 3c) (López-Maury et al. 2002).

Rre1 is a LuxR/NarL family response regulator identified in both PCC 6803 and PCC 7942. In PCC 7942, ChIP-chip assays show that Rre1 binds a proposed DNA-binding motif 5’-GTNCGGK-3’ (Kobayashi et al. 2017). Rre1 functions as an activator in PCC 7942; its regulon includes protein chaperones and type II sigma factor (*rpoD2*/*sigB*) (Fig. 3a) (Kobayashi et al. 2017). *In vitro* experiments have also demonstrated Rre1 oligomerization under oxidizing conditions, with thioredoxin restoring the monomeric state (Ibrahim et al. 2022). Thioredoxin redox status may provide a second redox input independent of Hik2 phosphotransfer, however, further experimental validation will be required to characterize this mechanism. 

DnaK2 and RpoD2 extend regulatory control downstream of Rre1. In PCC 6803, the Rre1 signal is amplified by RpoD2/SigB, which directs a broad regulon spanning oxidative stress, heat shock, and circadian programs (Imamura et al. 2003, Hakkila et al. 2014, Hakkila et al. 2019, Turunen et al. 2022). In PCC 7942, DnaK2 creates a negative feedback loop on Hik2 signaling: under low proteotoxic stress, free DnaK2 represses Hik2 phosphorylation of Rre1. When damaged proteins accumulate, DnaK2 is titrated away from Hik2, derepressing Rre1 phosphorylation and upregulating the chaperone regulon. The resulting increase in protein repair capacity restores DnaK2 availability, re-establishing repression (Fig. 3d) (Hasegawa et al. 2024).

### PQ redox inputs calibrate the circadian clock in PCC 7942

In cyanobacteria, redox regulation and circadian timekeeping are tightly coupled. Transcriptional programs activated at night maintain cellular redox homeostasis in the absence of photosynthetic electron flow (Diamond et al. 2017), and circadian time influences susceptibility to ROS damage (Tanaka et al. 2020). External electron transfer to the PQ pool under otherwise constant conditions is sufficient to entrain the circadian oscillator (Lu et al. 2014), establishing that the clock integrates PQ redox state directly.

**Figure 4:**
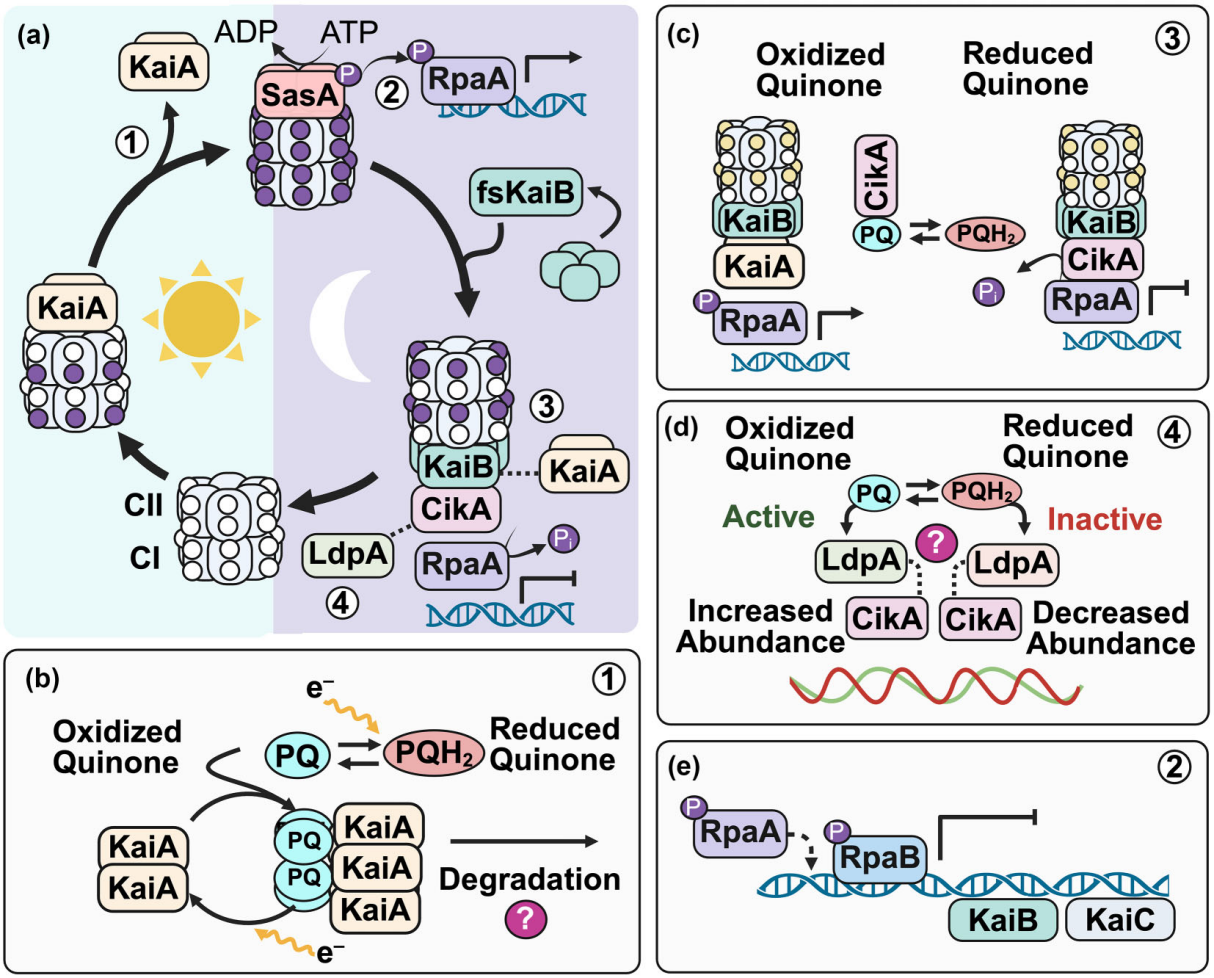
Redox inputs into the circadian clock KaiABC and output RpaA. (a) Redox regulation of the primary clock and circadian output, with the primary KaiABC circadian cycle model derived in PCC 7942 from a previous review by Swan et al. 2018. (b) KaiA binding oxidized quinones leads to dissociation from KaiC, and protein aggregation. It is hypothesized KaiA is then proteolytically degraded, KaiA abundance decreasing after dark. (c) CikA binding to oxidized quinones results in dissociation from the KaiBC complex, facilitating KaiA binding, which competes with CikA for a similar binding pocket on KaiB. (d) LdpA serves as another sensor of oxidized quinones through its bound [4Fe-4S] cluster. Oxidation activates LdpA, increasing intracellular CikA abundance. Reduced LdpA results in lower CikA concentrations and a phase shift in the circadian cycle. The mechanism by which LdpA controls CikA protein abundance is unknown. (e) RpaB binds the *kaiBC* operon promoter, inactivating it, preventing activation by RpaA, and halting the transcription-translation feedback loop (TTFL). Created with BioRender.com.

The KaiABC circadian clock, first described in the model cyanobacterium *S. elongatus* PCC 7942, functions as a post-translational oscillator. The phosphorylation state of the KaiC hexamer progresses through a ∼24-h cycle driven by opposing activities of KaiA and KaiB (Hayashi et al. 2004, Phong et al. 2013). Signal output from the oscillator proceeds through the histidine kinase SasA and the phosphatase CikA, which have opposing effects on the response regulator RpaA (Fig. 4a) (Gutu and O’Shea 2013, Markson et al. 2013). KaiA, CikA, and the iron-sulfur protein LdpA sense the redox state of the plastoquinone pool, coupling the oscillator to photosynthetic electron transport (Schmitz et al. 2000, Katayama et al. 2003, Ivleva et al. 2005, 2006). A second response regulator, RpaB, adds a further layer of control over clock output in PCC 7942 (Hanaoka et al. 2012, Espinosa et al. 2015, Piechura et al. 2017). This KaiABC core is broadly conserved across cyanobacteria, with individual species elaborating on it to differing degrees. The facultative heterotroph *Synechocystis* sp. PCC 6803, for example, carries additional KaiB and KaiC homologs together with a chimeric KaiA3, which assemble into a second KaiA3B3C3 oscillator interconnected with the canonical KaiA1B1C1 system and important for growth on glucose in the dark (Köbler et al. 2024).

Three clock-associated proteins sense and relay the redox state of the plastoquinone pool to the oscillator: KaiA and CikA through quinone-binding pseudoreceiver domains, and LdpA through a redox-sensitive iron-sulfur cluster. During the circadian day, KaiA stimulates KaiC autophosphorylation, and hyperphosphorylated KaiC recruits the histidine kinase SasA, which phosphorylates RpaA to activate dusk gene expression (Kim et al. 2008, Gutu and O’Shea 2013, Markson et al. 2013, Phong et al. 2013, Abe et al. 2015). At the onset of darkness, oxidized quinones bind the KaiA pseudoreceiver domain, triggering aggregation and dissociation from KaiC, which reinforces the transition to the dephosphorylation phase (Fig. 4b) (Kim et al. 2008, Wood et al. 2010, Kim et al. 2012). Oxidized quinones simultaneously bind the pseudoreceiver domain of the phosphatase CikA, disrupting its association with the KaiB-KaiC complex and reducing its phosphatase activity toward RpaA (Fig. 4c) (Schmitz et al. 2000, Ivleva et al. 2006). A third input operates through LdpA, whose [4Fe-4S] cluster senses PQ redox state and modulates intracellular CikA concentration: oxidized LdpA increases CikA abundance, while reduced LdpA lowers it and shifts circadian phase (Fig. 4d) (Katayama et al. 2003, Ivleva et al. 2005). Together, these three PQ-responsive inputs (KaiA, CikA, LdpA) calibrate both the timing of the oscillator and the magnitude of its transcriptional output through RpaA.

RpaB further modulates the circadian output of RpaA through two mechanisms (Fig. 4e). At the promoter level, RpaB binds HLR1 elements upstream of *rpaA, kaiBC*, and circadian sigma factors, several of which overlap RpaA target promoters, so that the two regulators compete for shared sites and gate the transcription-translation feedback loop (Hanaoka et al. 2012, Piechura et al. 2017). At the protein level, the RpaB receiver domain reduces RpaA∼P levels through a mechanism independent of DNA binding, as overexpression of the receiver domain alone is sufficient to attenuate RpaA phosphorylation (Espinosa et al. 2015). The molecular basis of this second interaction remains unresolved.

The quinone- and cluster-based sensing described above is not the only route to redox or environmental input among cyanobacterial clocks. In PCC 6803, the chimeric KaiA3 carries a genuine response-regulator receiver domain with a conserved phosphoacceptor aspartate, rather than the quinone-binding pseudoreceiver of canonical KaiA, implying that this oscillator responds to a distinct and still unidentified signal, potentially relayed by a cognate histidine kinase (Köbler et al. 2024).

In PCC 7942, PQ redox signals calibrate circadian timing through KaiA, CikA, and LdpA, with RpaB shaping the resulting transcriptional output. The converse is also true: the circadian clock actively maintains redox homeostasis during the night, when photosynthetic electron flow ceases. Redox cofactors cycle over the circadian period in constant light, dependent on the regulatory output of RpaA (Nishio et al. 2015, Tanaka et al. 2019). Phosphorylation of RpaA directly regulates the expression of over 150 genes in PCC 7942, including oxidative pentose phosphate pathway enzymes (*opcA, zwf, gnd*), NADPH recycling machinery (*pntAB, hox* operon), and glycogen utilization enzymes (*glgP, malQ*) (Markson et al. 2013, Piechura et al. 2017, Welkie et al. 2019). The RpaA target OpcA is further regulated post-translationally through thiol PTMs that allosterically control G6PDH activity on sub-second timescales, providing a rapid redox response layer that operates in parallel with circadian transcriptional control of *opcA* expression (Kim et al. 2026). Among the other downstream regulators activated by RpaA, several control redox-relevant programs, including overlap with the Rre1 proteostasis regulon through RpoD2/SigB (Kobayashi et al. 2017, Fleming and O’Shea 2018), the RpaB regulon via RpoD6 (Hanaoka et al. 2012, Markson et al. 2013), and RpoD5/SigC. TetR and SrrB have additionally been identified as high-centrality nighttime regulators that mediate RpaA’s indirect control over glycogen mobilization and redox metabolism in PCC 7942 (Johnson et al. 2025b).

## Thylakoid-localized sensing of photosystem metal cofactors in PCC 6803

**Figure 5:**
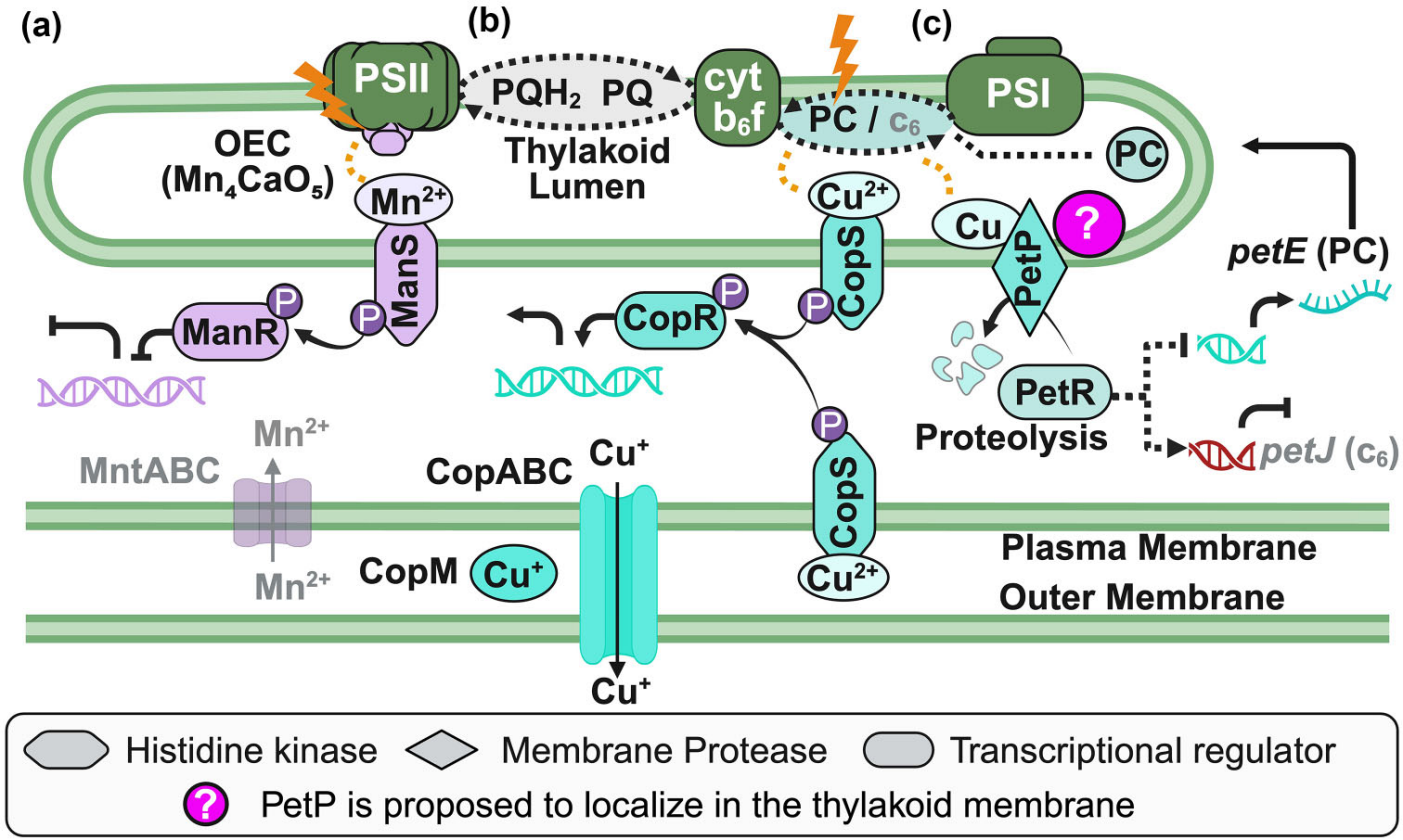
Two-component systems localized in the thylakoid membrane sense photosystem metal cofactors. (a) ManRS controls manganese homeostasis. The HK ManS, localized to the thylakoid membrane, functions as a sensor for Mn^2+^, the cofactor for the PSII oxygen-evolving complex (OEC, Mn_4_CaO_5_). Sensing Mn^2+^ (OEC disruption), ManS phosphorylates ManR, repressing the transcription of the Mn^2+^ ABC transporter operon (*mntCAB*). (b) CopRS controls copper homeostasis. CopS localizes to both the thylakoid and plasma membranes, sensing Cu^2+^ in the thylakoid lumen (released by plastocyanin degradation). Cu^2+^-bound CopS phosphorylates cytoplasmic CopR, which activates transcription of the copper resistance operons *copMRS* and *copBAC* (encoding CopM and the CopABC efflux pump shown at the plasma/outer membrane). (c) PetRP is predicted to localize to the thylakoid membrane. Sensing copper, PetP proteolytically degrades PetR, derepressing expression of *petE* (PC) and deactivating expression of *petJ* (cytochrome c6, Fe-heme cofactor). The result is expression of *petE* when copper is available and *petJ* when copper is absent. Created with BioRender.com.

Beyond sensing the redox state of electron carriers, thylakoid-localized two-component systems also monitor the availability of redox-active metal cofactors essential for photosystem function. The ManRS system senses free Mn^2+^ in the thylakoid lumen, reporting on the cofactor supply for the PSII oxygen-evolving complex (Mn_4_CaO_5_) (Shen 2015, Wang et al. 2022). The CopRS and PetRP systems sense copper, linking plastocyanin metalation status to transcriptional regulation of copper homeostasis and the cytochrome c_6_/plastocyanin switch (Giner-Lamia et al. 2012, López-Maury et al. 2012, García-Cañas et al. 2021). By localizing these sensors directly at the thylakoid membrane (Giner-Lamia et al. 2012, Wang et al. 2022), cyanobacteria couple luminal metal availability to transcriptional responses that maintain cofactor supply for photosynthetic electron transport (Fig. 5a–c) (Keren et al. 2002, Giner-Lamia et al. 2012, López-Maury et al. 2012, Zorina et al. 2016).

### ManRS: sensing luminal Mn^2+^ and integrity of the oxygen-evolving complex

The ManRS two-component system coordinates manganese homeostasis in cyanobacteria. The histidine kinase ManS localizes to the thylakoid membrane in PCC 7002 (Dahlgren et al. 2021) and PCC 6803 (Wang et al. 2022). In PCC 6803, the sensory domain faces the luminal side of the thylakoid membrane, suggesting a role in sensing Mn^2+^ derived from the OEC (Wang et al. 2022). Upon sensing Mn^2+^, ManS represses high-affinity manganese uptake (*mntCAB*) by phosphorylating ManR (Fig. 5a) (Yamaguchi et al. 2002). Simultaneously, ManR in PCC 6803 has been identified as a response regulator that undergoes a thiol-disulfide exchange reaction with thioredoxin *in vitro* (Kadowaki et al. 2015). Redox proteomics show increased oxidation of C154–located in the DNA-binding domain of ManR–during PSII inhibition by DCMU and under dark cultivation (Guo et al. 2014). It is hypothesized that this oxidation may promote ManR oligomerization and attenuate DNA binding, allowing photosynthetic redox signals to modulate manganese repression independently of metal availability (Kadowaki et al. 2015).

### CopRS: sensing luminal Cu^2+^ and integrity of plastocyanin

The CopRS system similarly detects the availability of a transition metal Cu^2+^ in the thylakoid lumen (Giner-Lamia et al. 2012, López-Maury et al. 2012). In PCC 6803, plasma membrane- and thylakoid membrane-localized CopS senses elevated Cu^2+^ in the periplasm and thylakoid lumen and phosphorylates CopR. In its phosphorylated state, CopR binds upstream and activates the expression of its own operon (*copMRS*) and the RND type copper efflux system (*copBAC*) (Giner-Lamia et al. 2012). By localizing to the thylakoid membrane, CopS can detect disruption of photosynthetic electron transport by sensing free luminal Cu^2+^, the cofactor of the soluble electron carrier plastocyanin. Blocking electron transfer from the plastoquinone pool to cytochrome b_6_f with DBMIB when cells are grown under normal copper concentrations triggers a decrease in plastocyanin levels and induction of the CopR regulon. Here the authors propose that the accumulation of oxidized plastocyanin resulting from blocked electron transport results in its degradation and the release of oxidized copper (Cu^2+^) where activation of the CopRS regulon prevents oxidative damage to photosystem complexes catalyzed by copper mediated Fenton chemistry (Fig. 5b) (Giner-Lamia et al. 2012, López-Maury et al. 2012, Giner-Lamia et al. 2014). Still the exact mechanism for thylakoid lumen copper detoxification remains unresolved, as CopBAC exports copper from the cytosol and periplasmic space where CopM functions to sequester copper in the periplasm and extracellularly.

### PetRP: proteolytic regulation of soluble e^–^ carrier

Similar to CopRS, PetRP is responsible for sensing copper. Unlike CopS, PetP regulates transcription through irreversible proteolysis of PetR, which controls the use of soluble electron carriers in response to copper availability. Under copper limitation, the transcriptional regulator PetR represses *petE* (plastocyanin) and activates *petJ* (cytochrome c_6_), favoring transcription of the iron cofactor soluble electron carrier. Copper binding directly activates the membrane protease PetP, which degrades PetR and enforces the plastocyanin-dependent state (García-Cañas et al. 2021). PetP is predicted to be membrane-associated and hypothesized to localize to the thylakoid (Fig. 5c), however, its precise subcellular localization has not yet been experimentally resolved or determined by proteomic mapping (Baers et al. 2019). Together with CopRS, PetRP enables layered control of copper-dependent electron flow.

## Cytosolic redox signaling and transcriptional regulation downstream of PSI

**Figure 6:**
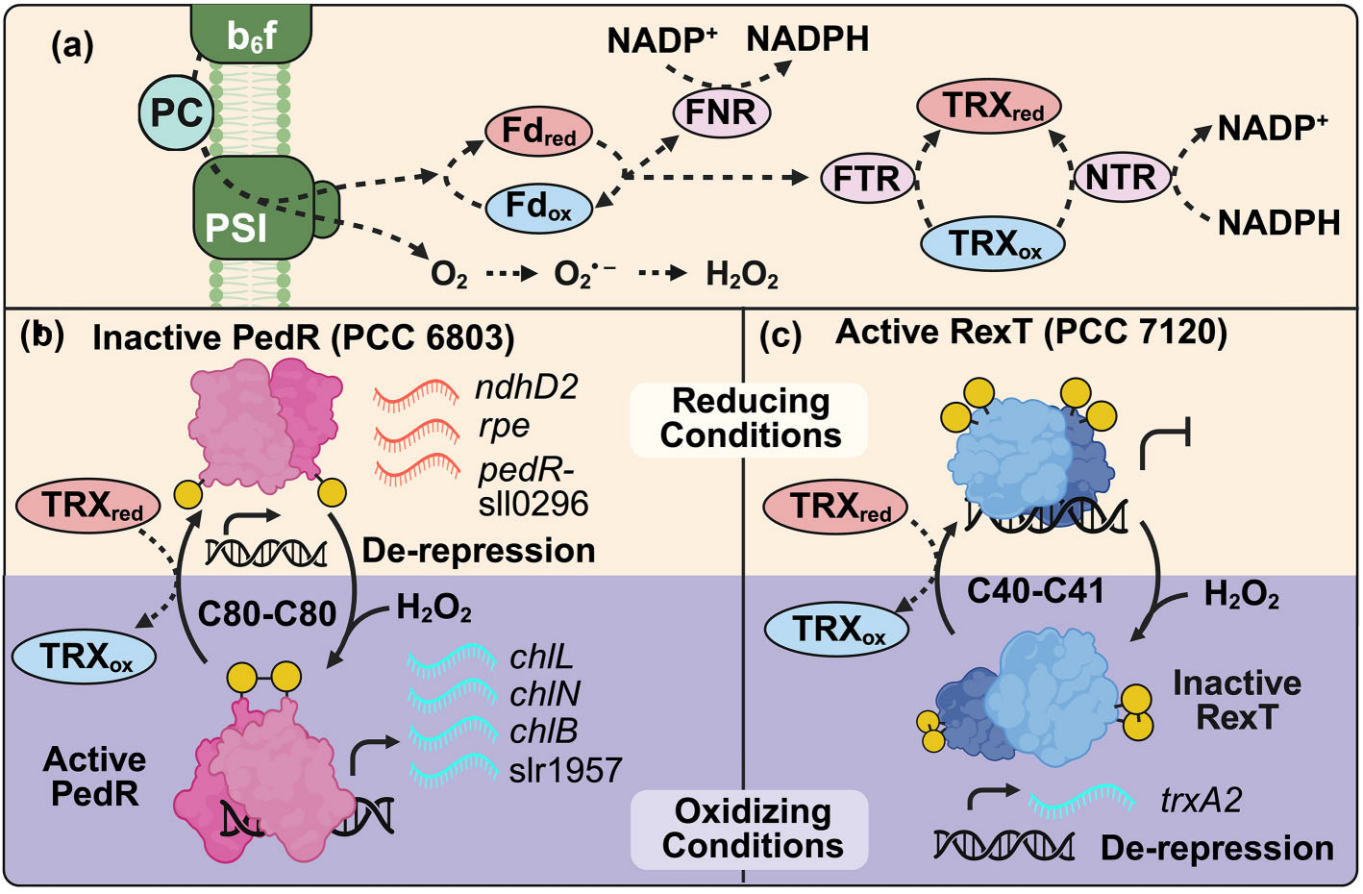
Non-metalated thiol switches PedR and RexT sense intracellular redox state maintained by PSI-derived electron flow. (a) Reducing equivalents from the PSI acceptor side set the intracellular redox state. PSI reduces ferredoxin (Fd_ox_ → Fd_red_), which donates electrons to ferredoxin-NADP^+^ reductase (FNR), generating NADPH, or to ferredoxin-thioredoxin reductase (FTR), which reduces thioredoxin (TRX_ox_ → TRX_red_); NADPH can also reduce TRX via NADPH-thioredoxin reductase (NTR). Reduced TRX then reduces disulfide bonds in regulatory proteins to control their activity. Electrons leaked from PSI to O_2_ generate superoxide (O_2_^–^) and ultimately H_2_O_2_ (lower branch), which drives PedR and RexT disulfide formation in panels b and c. (b) In PCC 6803, PedR binds DNA under oxidizing conditions, when an intermolecular disulfide forms between Cys80 of each monomer. DNA-bound PedR activates *chlL, chlN, chlB*, and *slr1957* and represses *ndhD2, rpe*, and the *pedR*-*sll0296* operon. Reduced TRX reduces the disulfide, releasing PedR and de-repressing the latter genes. (c) In PCC 7120, the RexT homodimer binds DNA under reducing conditions. Oxidation forms a vicinal intramolecular disulfide between Cys40 and Cys41, abolishing DNA binding and de-repressing *trxA2*. Created with BioRender.com.

In contrast to the membrane-localized two-component systems described above, one-component regulators sense cytosolic redox pools maintained by PSI-derived electron flow through ferredoxin (Fig. 6a). For thioredoxin-regulated factors such as PedR, the link to PSI has been demonstrated directly: inactivation of ferredoxin-thioredoxin reductase or NADPH-thioredoxin reductase abolishes the redox-dependent conformational change of PedR under high light (Nakamura and Hihara 2006, Horiuchi et al. 2010). The subsections below are organized by sensing chemistry, proceeding from non-metalated thiol switches to metalated thiol switches (FUR family regulators), Fe-S cluster sensors, and finally to glutathionylated nucleoid-associated proteins.

### Non-metalated thiol switches: PedR and RexT

#### PedR: control of chlorophyll biosynthesis and respiratory electron transport in PCC 6803

PedR provides the most direct link between PSI electron flow and transcriptional regulation in cyanobacteria. It binds a conserved DNA motif 5′-RWWTRGGYNNYY-3′, functioning as both an activator and a repressor, activating chlorophyll biosynthesis genes (*chlL, chlN, chlB, slr1957*) and repressing *ndhD2, rpe*, and its own operon (*pedR*–sll0296). PedR senses redox state via C80, which is responsible for PedR dimerization. Under low light (oxidizing conditions), PedR is an active DNA-binding disulfide-linked dimer, while high light (reducing conditions) promotes thioredoxin-dependent reduction by either TrxM or TrxX, triggering a conformational change and preventing DNA binding (Fig. 6b) (Nakamura and Hihara 2006, Horiuchi et al. 2010).

#### RexT: control of alternative thioredoxin expression in PCC 7120

RexT is a transcriptional repressor of the ArsR/SmtB family in *Anabaena* sp. PCC 7120 that regulates the thioredoxin gene *trxA2* in response to oxidative stress (Ehira and Ohmori 2012). It functions as a homodimer with DNA-binding residues R26 and K50 recognizing a palindromic binding motif 5′-ATTCG–N_15_–CGAAT-3′ (Li et al. 2022). Unlike canonical ArsR regulators (Morby et al. 1993), RexT does not bind metal ions; instead, it senses redox state via vicinal cysteine residues C40 and C41, which form an intramolecular disulfide bond upon oxidation. Under reducing conditions, RexT represses *trxA2*, while oxidation triggers derepression (Fig. 6c) (Ehira and Ohmori 2012).

### Metalated thiol switches: FUR family transcriptional regulators in PCC 7120

While PedR and RexT sense PSI acceptor side redox state exclusively through cysteine oxidation, the ferric uptake regulator (FUR) family integrates thiol chemistry with additional inputs: metal coordination, heme binding, and ROS-driven cofactor oxidation. Their transcriptional output therefore reflects a convergence of redox and metal signals rather than a function of thioredoxin reduction state (Lee and Helmann 2007, Fillat 2014). These proteins share a modular architecture with an N-terminal winged helix–turn–helix DNA-binding domain and a C-terminal metal-binding/dimerization domain. Variation in metal specificity, cysteine content, and regulatory site geometry enables FUR family members to function as graded sensors that scale transcriptional output with intracellular metal and redox status rather than acting as binary switches.

#### Fur (FurA): global iron homeostasis

Iron requirements are ∼10-fold higher in cyanobacteria than in typical non-photosynthetic heterotrophic bacteria (Keren et al. 2004, Shcolnick and Keren 2006). Iron is essential for all membrane-bound protein complexes involved in photosynthetic electron transport and for mobile electron carriers (Ferreira and Straus 1994). Diazotrophic cyanobacterial species such as PCC 7120 require additional iron due to high demands from nitrogen-fixing nitrogenase proteins (Richier et al. 2012). Despite these high iron requirements, iron is often limiting in aquatic environments, constraining cyanobacterial growth (Geider and La Roche 1994). Simultaneously, iron is linked to oxidative stress, in which free intracellular iron (Fe^2+^) can catalyze the formation of hydroxyl radicals (·OH) via Fenton reactions (Latifi et al. 2009). Efficient iron scavenging, incorporation, and storage are therefore critical processes that are regulated by the essential global iron homeostasis transcriptional regulator Fur (FurA) (Hernández et al. 2006b, Ghassemian and Straus 1996).

**Figure 7:**
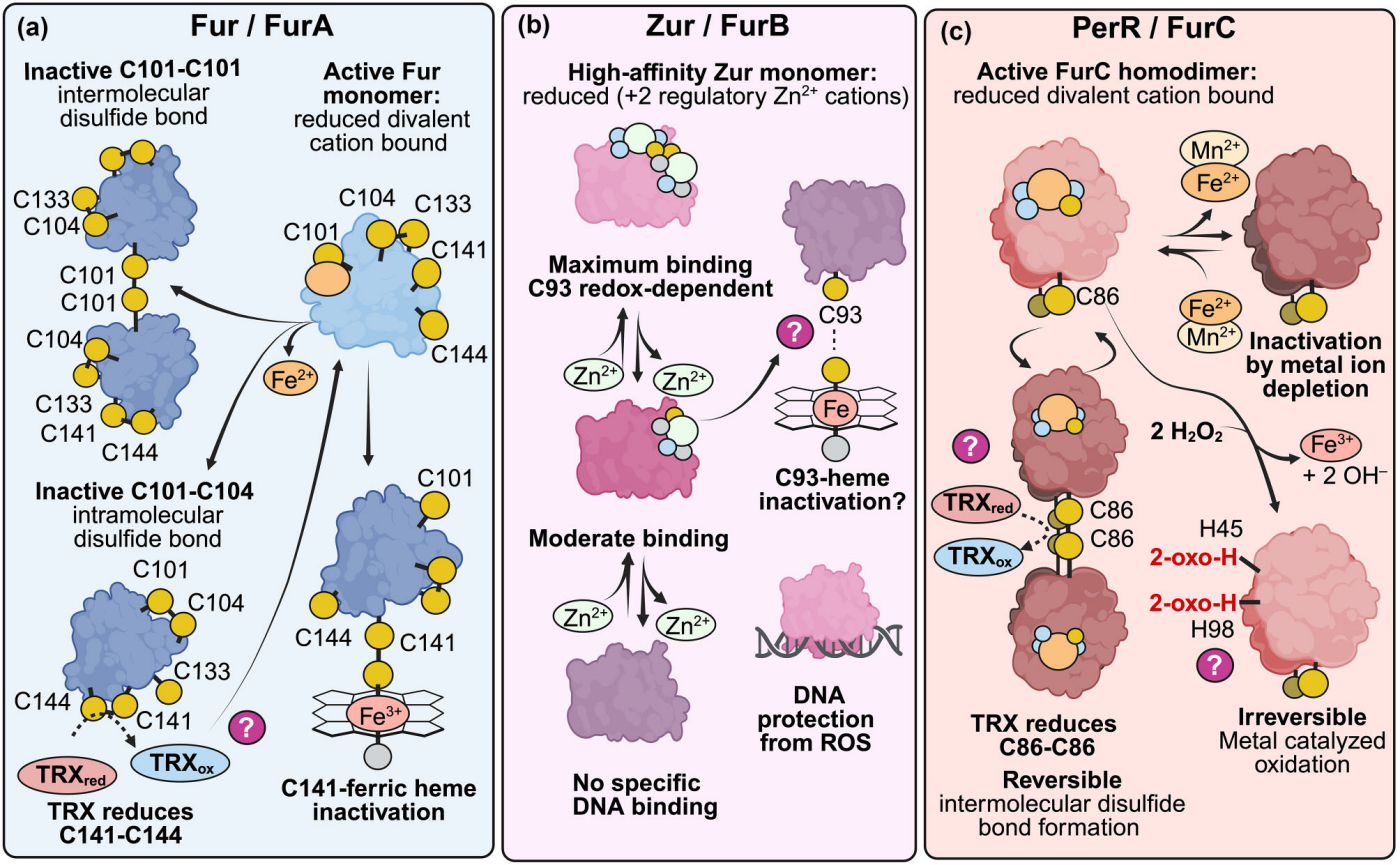
Redox control of FUR family protein regulation in *Anabaena* sp. PCC 7120. (a) Redox regulation mechanisms for Fur. Under iron replete reducing conditions Fur binds DNA, coordinating Fe^2+^ through the regulatory thiol (C101), with C104 and C133 forming an intramolecular disulfide bond. Under oxidizing conditions, thiol-disulfide interchange forms an intramolecular C101–C104 disulfide bond, which releases the metal cofactor and abolishes DNA binding. C101 can also form an intermolecular C101–C101 disulfide bond between monomers, producing disulfide-linked dimers which lose DNA binding affinity *in vitro*. TRX can reduce the disulfide bond between C141 and C144, hypothetically initiating a series of disulfide exchange reactions that free the regulatory thiol (C101). Independently, binding ferric heme (Fe^3+^) at C141 represses Fur DNA binding in a concentration-dependent manner. (b) Redox regulation mechanisms for Zur. DNA binding is governed by two regulatory Zn^2+^ binding sites, with the second site regulated by the redox state of C93. C93 may also be involved in coordinating heme as a direct ligand or proximal residue. Independently of Zur binding specific targets, *in vitro* studies demonstrate non-specific DNA binding by Zur is involved in the protection of DNA damage from ROS. (c) Redox regulation mechanisms for PerR. Iron-loaded PerR (FurC:Zn-Fe) binds DNA and represses its regulon; under iron limitation, PerR enters an activatable apo-state (top right) that recovers DNA binding upon iron repletion (Sarasa-Buisan et al. 2022a). Under mild oxidative stress, reversible intermolecular C86-C86 disulfide bond formation between two dimers produces a tetrameric structure that is inactive but reducible by thioredoxin. Under severe oxidative stress, when PerR is bound to Fe²⁺, metal-catalyzed oxidation (MCO) is proposed to irreversibly inactivate PerR; two of the Fe²⁺-coordinating histidine residues (H45, H98) structurally align with the residues that undergo MCO in *Bacillus subtilis*. Created with BioRender.com.

Fur activity is dependent on iron availability (Ghassemian and Straus 1996, Hernández et al. 2006a, Botello-Morte et al. 2016), cellular redox state (Hernández et al. 2006b, Lostao et al. 2010, Botello-Morte et al. 2016), heme (Pellicer et al. 2012), and 2-oxoglutarate (2-OG) levels (Guío et al. 2020). Fur regulation is mediated by five C-terminal cysteine residues (C101, C104, C133, C141, C144), four of which are arranged into two redox-active CXXC motifs (C101–C104 and C141–C144) (Botello-Morte et al. 2016). Unlike canonical FUR proteins, cyanobacterial Fur lacks a structural Zn^2+^ ion (Hernández et al. 2002), making these thiols redox-sensitive and allowing regulation through reversible intra- and intermolecular disulfide bond formation (Botello-Morte et al. 2016). The C101 residue is central to Fur activity, coordinating Fe^2+^ or Mn^2+^ under reducing conditions, with C133 forming a disulfide bond with C104. Under oxidative stress, the formation of an intramolecular disulfide bond (C101–C104) or an intermolecular disulfide bond (C101–C101) disrupts metal binding and inhibits DNA association (Fig. 7a). Independently, C141 functions as a heme sensor (Igarashi et al. 2008, Pellicer et al. 2012), where ferric heme suppresses Fur DNA binding in a concentration-dependent manner (Hernández et al. 2004). Reactivation of oxidized Fur is proposed to proceed via TrxA-mediated reduction of the C141–C144 disulfide bond, followed by intramolecular disulfide exchange that restores C101 metal-binding competency (Fig. 7a) (Guío et al. 2021).

In the active form, Fur recognizes the A/T-rich FUR box, functioning primarily as a repressor but also activating gene expression (González et al. 2012, González et al. 2014). In PCC 6803 (Riediger et al. 2021) and PCC 7942 (Ghassemian and Straus 1996), Fur is responsible primarily for iron uptake and storage. In contrast, FurA governs a wider set of genes in PCC 7120, simultaneously regulating pigment biosynthesis, cell morphology, and oxidative stress response (Hernández et al. 2007, González et al. 2010, 2012).

#### Zur (FurB): zinc homeostasis

Zinc is an essential micronutrient in cyanobacteria that plays a central role in redox biology by coordinating protein thiolates, acting in both redox-active and redox-inert roles depending on the protein site (Maret 2019). Zinc homeostasis is regulated by metallosensing transcription factors, including the FUR family repressor Zur (FurB), which has been shown to sense the cellular redox state to maintain zinc balance in PCC 7120 (Sein-Echaluce et al. 2018).

Zur integrates zinc (Kandari et al. 2021), heme (López-Gomollón et al. 2009, Sein-Echaluce et al. 2018), and redox signals (Sein-Echaluce et al. 2018) to regulate transcription. In PCC 7120, Zur exhibits a graded transcriptional response to intracellular zinc, binding DNA weakly when coordinated by a single regulatory Zn^2+^ ion and with high affinity upon binding a second regulatory Zn^2+^ (Sein-Echaluce et al. 2018). This transition is gated by the redox state of cysteine C93, which modulates zinc coordination at the second Zn^2+^ binding site and promoter-specific DNA affinity (Sein-Echaluce et al. 2018). Notably, C93 may also participate in heme binding, but is not essential for heme-dependent repression of Zur activity (Sein-Echaluce et al. 2018). These mechanisms facilitate sensing of zinc availability, redox state, and heme to control transcriptional programs that protect genomic integrity under oxidative stress (Fig. 7b).

Across cyanobacteria, Zur controls its own expression and the zinc ABC transporter (*znuABC*) (Novichkov et al. 2013). Zur has also been recognized for its contributions to oxidative stress response, motility, biofilm formation, and *fur* expression in PCC 6803 (Jin et al. 2025) and PCC 7120 (Olivan-Muro et al. 2023). Beyond its recognized role as a transcriptional regulator, Zur exhibits a secondary role at high intracellular concentrations, protecting DNA *in vitro* from both DNase I cleavage and hydroxyl-radical damage (López-Gomollón et al. 2009).

#### PerR (FurC): hydrogen peroxide detoxification

Superoxide (O_2_^–^) produced by the photosynthetic electron transport chain (Pospíšil 2016) is reduced to H_2_O_2_ by superoxide dismutase, requiring further detoxification by either peroxidase or catalase (Latifi et al. 2009). When H_2_O_2_ accumulates in the presence of iron, Fenton reactions with Fe²⁺ produce the broadly damaging hydroxyl radical (·OH) (Imlay 2003). To sense and detoxify H_2_O_2_, cyanobacteria employ the FUR family protein PerR (FurC).

PerR activity is modulated by redox signals and the ratio of divalent cations (Fe^2+^ to Mn^2+^) coordinated at the protein regulatory site. The metal cation bound at the regulatory site determines whether PerR can directly sense and respond to H_2_O_2_. When Fe^2+^ occupies the regulatory site, H_2_O_2_ drives metal-catalyzed oxidation (MCO), incorporating one oxygen atom per monomer and irreversibly inactivating the protein. In *Bacillus subtilis*, MCO oxidizes one of two histidine residues at the Fe^2+^ site, H37 or H91, to 2-oxo-histidine (Traoré et al. 2009). In PCC 7120, PerR residues H45 and H98 structurally align with these oxidized residues but require additional experimental validation (Fig. 7c) (Sarasa-Buisan et al. 2022a). Conversely, Mn^2+^ does not catalyze Fenton chemistry (Smethurst and Shcherbik 2021), thereby protecting PerR from MCO and tuning the stress response to the presence of free iron. Independent of the regulatory metal cation, mild oxidation can trigger oligomerization of PerR dimers to form tetramers and higher-order oligomers. Structural modelling suggests an intermolecular disulfide bond at C86 bridges the dimers in this assembly. This state is reversible and can be reduced by chemical reducing agents (DTT) (Sarasa-Buisan et al. 2022a). *In vivo*, reduction by thioredoxin is plausible given its role in reducing the paralog FurA (Guío et al. 2021).

When active, PerR functions primarily as a transcriptional repressor by binding the PerR box motif (Sarasa-Buisan et al. 2023). Studies in PCC 7120 demonstrate that PerR acts as a global regulator, controlling genes involved in H_2_O_2_ detoxification (Sevilla et al. 2019), carbon metabolism (Sarasa-Buisan et al. 2023), nitrogen metabolism (Sarasa-Buisan et al. 2022b), photosynthesis (Yingping et al. 2014), cell division (Yingping et al. 2014), and cell wall remodeling (Sarasa-Buisan et al. 2024).

### Iron-sulfur cluster sensors control cofactor biosynthesis

Rather than sensing thiol oxidation state directly, SufR and ChlR use iron-sulfur clusters as redox-responsive cofactors. Cluster integrity is sensitive to oxygen, oxidative stress, and iron availability, coupling cofactor biosynthesis to conditions that affect PSI function.

#### ChlR: control of chlorophyll and tetrapyrrole biosynthesis

ChlR is a MarR-type transcriptional activator that senses oxygen availability in PCC 6803 and PCC 7002 (Aoki et al. 2012, Ludwig et al. 2014). It functions as a homodimer that binds a palindromic 5′-TTMCC–N_3_/_4_–GGWAA-3′ motif to activate the tetrapyrrole biosynthesis operon (*chlA2-ho2-hemN*) under low-oxygen conditions (Aoki et al. 2012, Ludwig et al. 2014). ChlR directly senses oxygen via a [4Fe-4S] cluster coordinated by N-terminal cysteine residues (C18, C25) in PCC 6803 (Fujita et al. 2015). Here, the iron-sulfur cluster stabilizes the DNA-binding conformation under microoxic conditions, with oxygen degrading the cluster, causing dissociation from promoters and transcriptional inactivation (Aoki et al. 2012, Ludwig et al. 2014).

#### SufR: iron-sulfur cluster biogenesis

**Figure 8:**
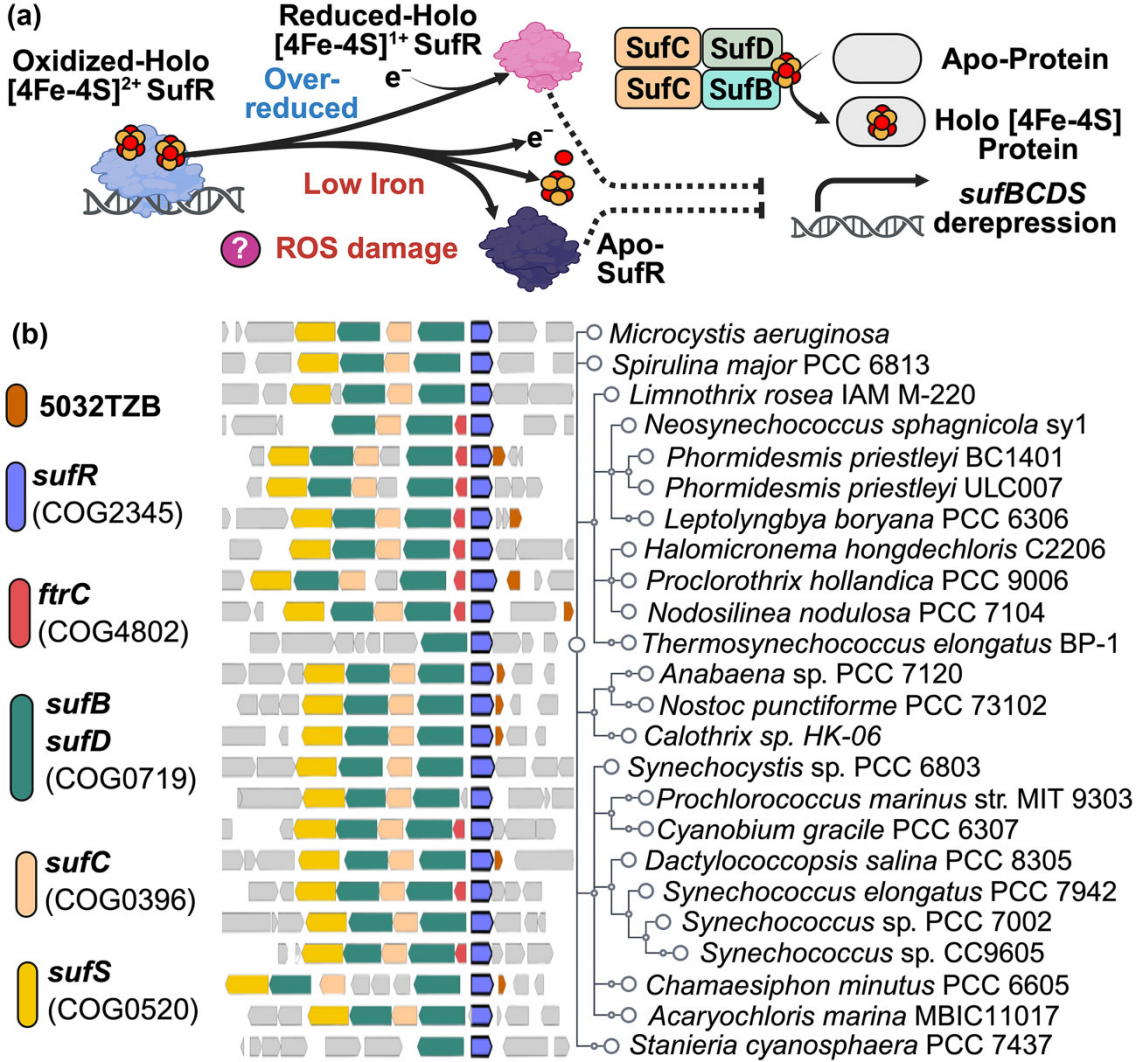
Iron-sulfur biogenesis and redox regulation in cyanobacteria. (a) Three physiological conditions converge on derepression of the *sufBCDS* operon. Under homeostatic conditions (top), oxidized holo-SufR ([4Fe-4S]^2+^) represses both *sufBCDS* and *sufR*. Over-reduction of the PSI acceptor side reduces the clusters to [4Fe-4S]^1+^, decreasing DNA-binding affinity, demonstrated *in vitro* by Shen et al. 2007. Iron limitation prevents cluster assembly, producing apo-SufR, supported by *in vivo* data from Wang et al. 2004. ROS may contribute to cluster loss either through direct damage or indirectly by oxidizing Fe^2+^ to Fe^3+^, depleting bioavailable iron, inferred by Wang et al. (2004). Derepression upregulates SUF machinery, which assembles new [4Fe-4S] clusters onto apo-proteins, including SufR itself, restoring repression (negative feedback loop). (b) Conservation of the *sufR*–*sufBCDS* genomic neighborhood across cyanobacteria. Two genes frequently co-localize with the SUF machinery: an uncharacterized ortholog (5032TZB = ENOG5032TZB) and ferredoxin-thioredoxin reductase subunit (*ftrC*), linking [Fe-S] cluster biogenesis to the thioredoxin relay. Created with BioRender.com.

SufR is a transcriptional repressor that regulates iron-sulfur cluster biogenesis, ensuring the supply of [Fe-S] cofactors required by photosynthetic complexes and redox-sensor proteins (Wang et al. 2004, Outten and Theil 2009). SufR functions as a homodimer coordinating two [4Fe-4S] clusters through conserved cysteine residues (C164, C171, C206) (Shen et al. 2007). In the oxidized [4Fe-4S]^2+^ state, SufR homodimers bind the inverted repeat motif 5'-CAAC-N_6_-GTTG-3', repressing the *sufBCDS* operon and its own transcription (Wang et al. 2004, Shen et al. 2007). Reduction of the clusters to the [4Fe-4S]^1+^ state significantly decreases DNA-binding affinity, derepressing iron-sulfur cluster biogenesis (Fig. 8a) (Shen et al. 2007). *In vivo, sufBCDS* expression is elevated under both oxidative stress and iron limitation (Wang et al. 2004), conditions that impair cluster assembly or integrity. SufR is also implicated in the transcriptional regulation of ferredoxin-thioredoxin reductase subunit (*ftrC*), which is co-transcribed with the *suf* operon in PCC 7942 (Johnson et al. 2025c, Vijayan et al. 2011). Comparative genomics across cyanobacteria demonstrates that *ftrC* commonly co-occurs in the genomic neighborhood of *sufBCDS* (Fig. 8b) (Botas et al. 2022) linking [Fe-S] cluster supply to the ferredoxin-thioredoxin relay described above.

### Glutathionylation of nucleoid proteins exerts global regulatory control in PCC 6803

**Figure 9:**
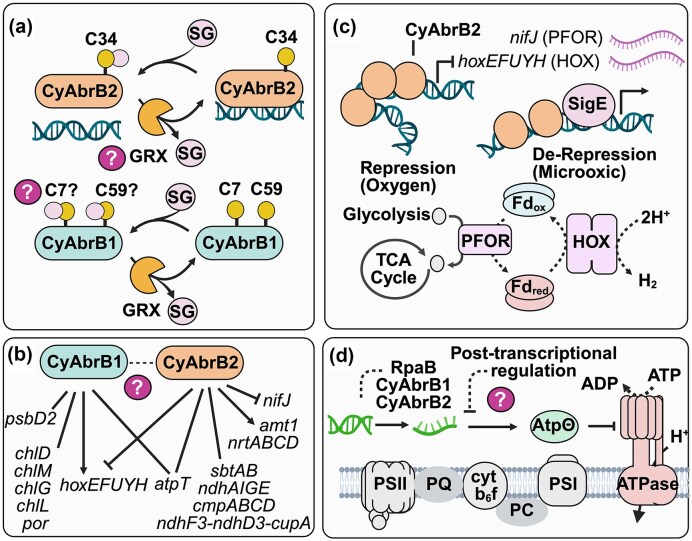
Transcriptional regulation by nucleoid-associated proteins in PCC 6803. (a) Glutathionylation of the CyAbrB regulators. Glutathionylation of C34 represses DNA binding of CyAbrB2 to the *hox* operon *in vitro*. The modification is reversible by thiol reduction (DTT) and is likely dependent on glutaredoxin (GRX) *in vivo*. CyAbrB1 possesses two cysteine residues (C7 and C59) and can similarly be modified by glutathionylation, but the residue modified has not been characterized. (b) CyAbrB1 regulates chlorophyll biosynthesis genes (*chlD, chlM, chlG, chlL*, and *por*), the PSII D2 subunit gene (*psbD2*), and the ATPase regulator gene (*atpT*). CyAbrB1 also activates the *hox* operon ([NiFe] bidirectional hydrogenase*, hoxEFUYH*). In contrast, CyAbrB2 represses transcription of the *hox* operon and *nifJ* (pyruvate:ferredoxin oxidoreductase, PFOR), and activates nitrogen metabolism genes (*amt1, nrtABCD*). CyAbrB2 also binds upstream of *atpT* and modulates the transcription of carbon metabolism genes (*sbtAB, ndhAIGE, cmpABCD, ndhF3-ndhD3-cupA*). (c) CyAbrB2 regulates transcription by influencing chromosome topology. Microoxic conditions result in derepression of the *hox* and *nifJ* operons, which are transcribed in coordination with sigma factor SigE. PFOR couples the conversion of pyruvate to acetyl-CoA with ferredoxin reduction (Fd_ox_ → Fd_red_), while HOX oxidizes ferredoxin (Fd_red_ → Fd_ox_) and dissipates reducing power through hydrogen production. (d) Expression of *atpT* is controlled in part by CyAbrB1, CyAbrB2, and RpaB, but primarily through an unknown post-transcriptional mechanism. AtpΘ, the protein product of *atpT*, represses F_0_F_1_ ATP synthase to prevent ATP hydrolysis resulting from a weak proton motive force. Created with BioRender.com.

Unlike traditional regulators, the nucleoid-associated proteins CyAbrB1 and CyAbrB2 regulate transcription at both the levels of individual promoters and global chromosomal organization (Kariyazono and Osanai 2024), with CyAbrB1 being essential (Ishii and Hihara 2008). In PCC 6803, it has been proposed that CyAbrB proteins are regulated by glutathionylation, coupling their activity to the GSH/GSSG ratio and thereby sensing a distinct cellular redox pool (Fig. 1c) (Sakr et al. 2013). Glutathionylation of the conserved cysteine C34 on CyAbrB2 derepresses the [NiFe] bidirectional hydrogenase (*hox* operon) and enables electron dissipation through hydrogen production as a redox escape valve (Sakr et al. 2013). CyAbrB1 is similarly glutathionylated *in vitro*, although the modification has not been localized to either of its two cysteine residues C7 or C59 (Fig. 9a) (Sakr et al. 2013, UniProt Consortium 2025). CyAbrB1 functions as an activator rather than repressor of the *hox* operon (Oliveira and Lindblad 2008) and interacts with CyAbrB2 at the protein level (Yamauchi et al. 2011). How these proteins integrate their regulatory inputs remains unclear. High-throughput proteomics further identifies that CyAbrB1 and CyAbrB2 are phosphorylated during recovery from chlorosis (Spät et al. 2023), further complicating their post-translational regulation.

The CyAbrB regulons are broad and overlapping, encompassing carbon (Lieman-Hurwitz et al. 2009, Orf et al. 2016, Hishida et al. 2024), nitrogen (Ishii and Hihara 2008), and energy metabolism (Song et al. 2022b, Kariyazono and Osanai 2024, Yu et al. 2026) (Fig. 9b). ChIP-seq studies show CyAbrB2 binds AT-rich sequences covering over 15% of the PCC 6803 genome, where it represses transcription by interfering with sigma factor SigE binding and modifying chromosome topology (Kariyazono and Osanai 2024). Under microoxic conditions, this repression is relieved, enabling SigE-dependent activation of the *hox* and *nifJ* (pyruvate:ferredoxin oxidoreductase, PFOR) operons (Fig. 9c). CyAbrB1 additionally regulates carbohydrate metabolism (Hishida et al. 2024) and chlorophyll biosynthesis genes (Yu et al. 2026). CyAbrB1, CyAbrB2, and RpaB also bind the *atpT* promoter (Song et al. 2022b). The protein product, AtpΘ, prevents ATP hydrolysis by the F_0_F_1_ ATP synthase under low-energy conditions. Beyond these regulators, the primary mechanism controlling *atpT* expression occurs at the post-transcriptional level: the abundance and stability of the *atpT* mRNA depend on the redox state of the photosynthetic and respiratory electron transport chain (Fig. 9d) (Song et al. 2022a,b).

## Redox control of gene expression beyond transcriptional initiation

The regulatory systems discussed above identify photosynthetic electron flow and the redox state of cytoplasmic pools downstream of PSI as key signals controlling transcriptional initiation in cyanobacteria. Transcription, however, is only one level of control between a gene and its protein product. Redox perturbations simultaneously affect RNA processing, translation, and metabolic flux, meaning that transcriptional responses alone provide an incomplete picture. In this section, we examine how redox state shapes a transcript’s lifecycle through post-transcriptional and translational checkpoints, and how redox-driven changes in metabolism reprogram transcriptional regulation (Fig. 1e–g).

### Redox regulation of the transcript lifecycle in PCC 6803

Reduction of the plastoquinone pool induces transcription of the DEAD-box RNA helicase CrhR (Kujat and Owttrim 2000, Ritter et al. 2022). In PCC 6803, CrhR remodels RNA secondary structure and associates with both the polysome and the RNA degradosome (Rosana et al. 2016), functioning as a checkpoint regulating transcript translation or degradation. CrhR abundance is coupled to photosynthetic electron flow through transcriptional induction and redox-dependent mRNA stabilization (Kujat and Owttrim 2000); consequently, a more reduced plastoquinone pool yields greater capacity for post-transcriptional RNA remodeling. CrhR RNA binding is further dependent on a surface accessible C371 residue, a potential candidate for redox regulation (Bilger et al. 2024).

Redox signals also regulate translation directly. Oxidation of C122 in the ribosome assembly GTPase EngA inactivates ribosome assembly under high light (Llop et al. 2023). More broadly, structural proteomics in PCC 7942 shows that ribosomal subunits undergo widespread conformational remodeling within 30 min of a light transition, independent of changes in protein abundance (Sarkar et al. 2025), suggesting that the redox shift accompanying altered photosynthetic electron flow extends its influence on ribosome function beyond EngA inactivation alone. Oxidation of C105 and C242 in elongation factor EF-G (Kojima et al. 2009) and C82 in EF-Tu (Yutthanasirikul et al. 2016) suppresses D1 protein synthesis and impairs PSII repair during photoinhibition (Nishiyama et al. 2001). EF-G and EF-Tu are reactivated by thioredoxin, linking translational capacity directly to PSI-derived electron flow. Conditional TrxA depletion arrests protein synthesis and redirects carbon and nitrogen into storage polymers (Mallén-Ponce et al. 2024), underscoring that thioredoxin-dependent redox control extends well beyond transcriptional initiation.

### Metabolic transduction of redox signals to transcription in PCC 6803

Metabolic pathways buffer reducing power generated by photosynthesis and feed back to transcriptional regulation. Cyanobacteria dissipate excess reducing power through several electron sinks, in addition to carbon fixation. These include nitrate reduction (Klotz et al. 2015), sucrose biosynthesis (Santos-Merino et al. 2021, Muth-Pawlak et al. 2024), the flavodiiron Flv1/Flv3 Mehler-like reaction (Allahverdiyeva et al. 2013), and photorespiratory 2-phosphoglycolate metabolism (Eisenhut et al. 2008), where relative contributions depend on carbon availability and light regime (Allahverdiyeva et al. 2011). The collective capacity of these sinks determines the PQ pool reduction state and ROS production, which feed back to the transcriptional and translational mechanisms described above.

A central metabolite linking redox state to transcription is 2-oxoglutarate (2-OG), which sits at the intersection of the TCA cycle and nitrogen assimilation in cyanobacteria (Muro-Pastor et al. 2001). 2-OG accumulation signals nitrogen limitation and is sensed by the transcriptional regulators NtcA and NdhR (CcmR) (Jiang et al. 2018) as well as the signal transduction protein PII (Forchhammer and Selim 2020). Critically, hydrogen peroxide lowers intracellular 2-OG levels (Robles-Rengel et al. 2019), meaning that oxidative stress can mimic a nitrogen-replete signal and reprogram NtcA-dependent transcription independently of actual nitrogen status. Metabolic sink capacity thus shapes redox poise, redox poise modulates 2-OG, and 2-OG feeds back to reprogram transcription, closing a loop between electron transport and transcriptional output. How oxidative stress depletes 2-OG, whether through direct chemical oxidation (Mailloux et al. 2009), inhibition of isocitrate dehydrogenase (Domínguez-Martín et al. 2014), or accelerated consumption through alternative metabolic routes (Muro-Pastor et al. 2005), remains to be determined.

## Redox regulation in biotechnology

The regulatory architecture described above has direct implications for engineering cyanobacteria as cell factories. Any intervention that alters electron flow, cofactor balance, or photoprotection will engage the transcriptional regulatory systems described in this review, yet current approaches do not account for the coordinated regulatory responses these strategies elicit.

Engineering metabolic electron sinks can boost photosynthetic efficiency (Santos-Merino et al. 2021), redirect metabolic flux (Dan et al. 2022, Zhang et al. 2023), and improve electron flow toward target products (Appel et al. 2020, Kanygin et al. 2020), while deleting photoprotection mechanisms can prevent electron dissipation and enhance yield (Thiel et al. 2019). Without knowing how the cell will respond to these perturbations, interventions risk eliciting compensatory regulatory responses, as illustrated by ROS accumulation in free fatty acid-producing strains (Ruffing 2013) and chlorosis in high light following deletion of the flavodiiron proteins Flv1/3 (Selão et al. 2020). The regulators characterized in this review offer potential intervention points to mitigate unintended regulatory responses and to steer cells toward a more productive metabolic state (Table 2).

**Table 2:** A subset of functional gene sets controlled by redox-dependent transcriptional regulators in cyanobacteria.

Complex/pathway	Regulated genes	TF	Strain	References
**Linear photosynthesis**				
Photosystem II (PSII)	*psbD1, psbC*	Fur/FurA	PCC 7120	Leonhardt and Straus 1994
	*psbA1, psbB*	RpaB	PCC 7942, PCC 6803	Piechura et al. 2017, Riediger et al. 2019
	*psbD2*	CyAbrB1	PCC 6803	Yu et al. 2026
Cytochrome b_6_f (B_6_f)	*petB, petC1*	RpaB	PCC 7942, PCC 6803	Piechura et al. 2017, Riediger et al. 2019
Photosystem I (PSI)	*psaA, psaD, psaE, psaI, psaK1*	RpaB	PCC 7942, PCC 6803	Piechura et al. 2017, Riediger et al. 2019
	*psaK*	Fur/FurA	PCC 7120	González et al. 2014
Phycobilisome (PBS)	*cpcB, cpcG1, apcE*	RpaB	PCC 7942, PCC 6803	Piechura et al. 2017, Riediger et al. 2019
Soluble electron carrier cytochrome b_6_f → PSI	*petE* (PC), *petJ* (c_6_)	PetR	PCC 6803	García-Cañas et al. 2021
**Intracellular redox (PSI acceptor side)**				
Ferredoxin-thioredoxin reductase (FTR)	*ftrC*	SufR	PCC 7942	Novichkov et al. 2013, Johnson et al. 2025c
NADPH-thioredoxin reductase (NTR)	*ntrC*	PerR/FurC	PCC 7120	Sarasa-Buisan et al. 2023
Thioredoxin	*trxA2*	RexT	PCC 7120	Ehira and Ohmori 2012
Flavodoxin	*isiB*	Fur/FurA	PCC 7120	Hernández et al. 2006a
Pyridine nucleotide transhydrogenase (PNT)	*pntA1/2, pntB*	RpaA	PCC 7942	Markson et al. 2013
Bidirectional [NiFe]-hydrogenase (HOX)	*hoxUYHW-hypAB*	RpaA	PCC 7942	Vijayan et al. 2011, Markson et al. 2013, Piechura et al. 2017
	*hoxEFUYH*	CyAbrB1, CyAbrB2	PCC 6803	Sakr et al. 2013
**Respiration / oxidative phosphorylation**				
aa3-type cytochrome c oxidase (COX)	*coxBAC*	Fur/FurA	PCC 7120	González et al. 2014
	*ctaAB, ctaCDE* (or *coxBAC*)	RpaA	PCC 7942	Vijayan et al. 2011, Piechura et al. 2017
cbb3-type cytochrome c oxidase (Cbb_3_)	*ccoON*	RpaA	PCC 7942	Vijayan et al. 2011, Piechura et al. 2017
type-I NAD(P)H dehydrogenase (NDHI)	*ndhD2*	PedR	PCC 6803	Nakamura and Hihara 2006
	*ndhD1, ndhO*	RpaA	PCC 7942	Markson et al. 2013, Piechura et al. 2017
	*ndhF4, cupB, ndhM*	RpaB	PCC 7942	Vijayan et al. 2011, Piechura et al. 2017
	*ndhF3, ndhD3, cupA*	CcmR	PCC 6803	Figge et al. 2001
		CyAbrB2	PCC 6803	Orf et al. 2016
	*ndhAIGE*	CyAbrB2	PCC 6803	Orf et al. 2016
type-II NAD(P)H dehydrogenase (NDHII)	*ndbA*	RpaB	PCC 7942, PCC 6803	Piechura et al. 2017, Riediger et al. 2019
	*ndbC*	RpaA	PCC 7942	Piechura et al. 2017
F_0_F_1_ ATP synthase (ATPase)	*atpT*	CyAbrB1, CyAbrB2, RpaB	PCC 6803	Song et al. 2022b
**Cellular maintenance/defense/repair**				
Proteostasis	*groES, groEL1, groEL2, dnaK2, htpG*	Rre1	PCC 7942	Kobayashi et al. 2017
PSII repair/photoprotection	*ftsH2/3, hliA/C, nblA*	RpaB	PCC 7942, PCC 6803	Piechura et al. 2017, Riediger et al. 2019
H_2_O_2_ detoxification	*prxA, srxA, ahpC, CGT3, alr4404*	PerR/FurC	PCC 7120	Sevilla et al. 2019
PSI low iron stress	*isiA*	Fur/FurA	PCC 7120	Leonhardt and Straus 1994
Circadian clock	*kaiBC*	RpaA, RpaB	PCC 7942	Markson et al. 2013, Piechura et al. 2017
**Central carbon metabolism**				
Pentose phosphate pathway	*opcA, zwf*	RpaA	PCC 7942	Markson et al. 2013, Piechura et al. 2017
Glycolysis/gluconeogenesis	*gap, fbp1*	RpaA	PCC 7942	Markson et al. 2013, Piechura et al. 2017
Glycogen metabolism	*glgP, malQ*	RpaA	PCC 7942	Markson et al. 2013, Piechura et al. 2017
Photorespiration	*gcvP*	RpaB	PCC 7942, PCC 6803	Piechura et al. 2017, Riediger et al. 2019
Pyruvate → Acetyl-CoA	*nifJ*	CyAbrB2	PCC 6803	Kariyazono and Osanai 2024
**Nitrogen metabolism**				
Nitrate/nitrite reduction	*nirA, nirB, narB*	NtcA	PCC 7942	Vázquez-Bermúdez et al. 2002b
GS-GOGAT pathway	*glnA, glnN*	NtcA	PCC 7942	Sauer et al. 2000, Vázquez-Bermúdez et al. 2002b
**Cofactor biosynthesis**				
Tetrapyrrole biosynthesis	*chlA2-ho2-hemN*	ChlR	PCC 6803, PCC 7002	Aoki et al. 2012, Ludwig et al. 2014, Fujita et al. 2015
	*hemB, hemC, hemK, hemH1, hemH2, ho1*	Fur/FurA	PCC 7120	González et al. 2012
[Fe–S] cluster biosynthesis	*sufBCDS*	SufR	PCC 6803	Wang et al. 2004
Carotenoid biosynthesis	*crtP/pds*	RpaB	PCC 7942, PCC 6803	Piechura et al. 2017, Riediger et al. 2019
	*crtP/pds, crtB*	RppA	PCC 6803	Yu et al. 2025
Chlorophyll-a biosynthesis	*chlG, chlH*	RppA	PCC 6803	Yu et al. 2025
	*chlL, chlN, chlB*	PedR	PCC 6803	Nakamura and Hihara 2006
	*chlD, chlM, chlG, chlL, por*	CyAbrB1	PCC 6803	Yu et al. 2026
	*chlH, chlB*	RpaB	PCC 7942	Piechura et al. 2017
**Transporters & metallophores**				
HME-RND exporter (Cu)	*copBAC*	CopR	PCC 6803	Giner-Lamia et al. 2012
ABC transporter (Mn)	*mntCAB*	ManR	PCC 6803	Yamaguchi et al. 2002
ABC transporter (Ni)	*nrsBACD*	RppA	PCC 6803	López-Maury et al. 2002
ABC transporter (Zn)	*znuAB, znuC*	Zur/FurB	PCC 7120	Olivan-Muro et al. 2023
ABC transporter (Zn)	*znuAB*	Fur/FurA	PCC 7120	González et al. 2014
ABC transporter (Fe^3+^) dicitrate	*fecBCDE*	Fur/FurA	PCC 7120	González et al. 2014
Siderophore biosynthesis	all2649–2641	Fur/FurA	PCC 7120	González et al. 2012
TonB-dependent receptors (haem, ferrichrome)	alr3242, all1101, all2610	Fur/FurA	PCC 7120	González et al. 2012
Na^+^/H^+^ antiporter	*mnh* operon	CcmR	PCC 6803	Figge et al. 2001
High affinity CO_2_ transporter	*sbtAB*	CcmR	PCC 6803	Figge et al. 2001
		CyAbrB2	PCC 6803	Orf et al. 2016
Ammonium transporter	*amtB*	RpaA, NtcA	PCC 7942	Paz-Yepes et al. 2007
	*amt1*	NtcA	PCC 7942	Vázquez-Bermúdez et al. 2002a
		CyAbrB2	PCC 6803	Ishii and Hihara 2008
Nitrate/nitrite ABC transporter	*nrtABCD*	NtcA	PCC 7942	Vázquez-Bermúdez et al. 2002b
		CyAbrB2	PCC 6803	Ishii and Hihara 2008
Cyanate ABC transporter	*cynABDS*	NtcA	PCC 7942, PCC 6803	Harano et al. 1997
Bicarbonate ABC transporter	*cmpABCD*	CyAbrB2	PCC 6803	Orf et al. 2016
**Regulatory**				
Type II sigma factor	*rpoD3/sigD*	RpaB	PCC 7942, PCC 6803	Piechura et al. 2017, Riediger et al. 2019
	*rpoD2/sigB*	Rre1	PCC 7942	Kobayashi et al. 2017
	*rpoD5/sigC*	RpaA, RpaB	PCC 7942	Markson et al. 2013, Piechura et al. 2017, Johnson et al. 2025c
	*rpoD6*	RpaA, RpaB	PCC 7942	Markson et al. 2013, Piechura et al. 2017
Type III sigma factor	*sigF1*	RpaB	PCC 7942	Piechura et al. 2017
	*sigF2*	RpaA, RpaB	PCC 7942	Markson et al. 2013, Piechura et al. 2017

Targets are grouped by cellular function. For each gene set, the controlling regulator, the model cyanobacterium in which the interaction was characterized, and the validating study are listed.

Cytosolic one-component regulators offer targeted engineering entry points for tunable control of photosystem electron flow. In PCC 7120, RexT controls the expression of alternative thioredoxin *trxA2* (Li et al. 2022). Recent evidence suggests that SufR regulates *ftrC* in PCC 7942, linking iron-sulfur cluster biogenesis to the ferredoxin-TRX relay (Johnson et al. 2025c). PerR regulates *ntrC*, modulating the flow of electrons from NADP(H) to TRX in PCC 7120 (Sarasa-Buisan et al. 2023).

Alternatively, two-component systems offer global regulatory control over the expression of photosystems (NblS-RpaB), pigment biosynthesis (Hik2-RppA), and the circadian regulon (SasA/CikA-RpaA). In PCC 7942 engineered for sucrose export, circadian state alone produced a three-fold difference in sucrose productivity through coordinated suppression of glycogen synthesis and cell division alongside upregulation of glycogen degradation, the oxidative pentose phosphate pathway, and alternative electron flow (Gilliam et al. 2025). That the circadian oscillator can be entrained by electrochemically manipulating the plastoquinone redox state (Lu et al. 2014) raises the possibility that favorable metabolic states could be induced without genetic modification.

## Concluding remarks

Regulatory systems respond to changes in photosynthetic electron flow through redox and metal sensing in the cytoplasm and at the thylakoid, converging on the expression of genes for photosystem complexes, pigment and cofactor biosynthesis, and metal homeostasis. Yet critical questions regarding core mechanisms and the conservation of these mechanisms across cyanobacteria remain unanswered. Across studies, redox regulation mechanisms are only defined in a small subset of cyanobacteria: PCC 6803, PCC 7942, PCC 7120, and PCC 7002. Redox-dependent regulatory systems such as NblS-RpaB, Hik2-Rre1, CyAbrB2, and Fur are strongly conserved across cyanobacteria (Bohutskyi et al. 2026, Johnson et al. 2026), with Hik2 conserved in chloroplasts of land plants (Ibrahim et al. 2020), and RpaB conserved in chloroplasts of algae (Riediger et al. 2018). Other regulators, such as RexT, are less conserved, largely defined within the order *Nostocales* with only 11 homologs identified in the orders *Synechococcales* and *Oscillatoriales* (Li et al. 2022). Conserved regulatory systems can nonetheless vary in their transcriptional regulons and sensing mechanisms across homologs.

The FUR family regulators illustrate this variation. In PCC 7120 they act as global regulators (González et al. 2012, Olivan-Muro et al. 2023, Sarasa-Buisan et al. 2023), with Fur (FurA) and Zur (FurB) binding DNA as monomers rather than the dimers typical of the canonical proteins (Botello-Morte et al. 2016, Sein-Echaluce et al. 2018, Fillat 2014). In unicellular cyanobacteria, their oligomerization patterns have yet to be resolved, and these regulators are largely thought to control localized regulons (Novichkov et al. 2013). In PCC 6803, recent studies have started to expand the known scope of Fur (FurA) (Riediger et al. 2021, Liu et al. 2024) and Zur (FurB) (Jin et al. 2025). Similarly, the regulon of RpaB is only partially conserved between PCC 7942 and PCC 6803 (Riediger et al. 2019). This conserved regulon includes photoprotection mechanisms and the sigma factor *rpoD3*. Still, differences in the regulon affect RpaB regulatory control and give rise to distinct transcriptional feedback mechanisms, mediated by sRNA PsrR1 in PCC 6803 and an RpaB homolog SrrA in PCC 7942. Further experimental and computational work will clarify how broadly these regulons are conserved across the cyanobacteria.

Still, many mechanisms remain unresolved even in the model systems in which they have been defined. These include mechanisms for sensing the oxidation state of PQ: how Hik2 responds to PQ oxidation, how KaiA is degraded upon sensing oxidized PQ, and how LdpA controls CikA abundance under the same conditions. Other unresolved mechanisms include whether the PetP protease is localized to the thylakoid membrane and how multiple PTMs may coordinate CyAbrB1 and CyAbrB2 to control DNA binding and chromosome conformation. Perhaps most significant is how NblS senses the Q_A_ redox state and how this signal is transduced to RpaB to control gene transcription. That thioredoxin reduces multiple response regulators (ManR, RpaA, RpaB, Rre1) raises the question of whether cytoplasmic redox pools can bypass kinase-mediated phosphotransfer, introducing regulatory crosstalk not captured by canonical two-component signaling models.

The systems characterized to date likely represent only a fraction of the redox-responsive regulators encoded in cyanobacterial genomes. Candidates including PrqR, PlmA, RbcR, PfsR, and CheY family proteins show evidence of redox-dependent regulation but lack mechanistic characterization (Babykin et al. 2003, Ansong et al. 2014, Sadler et al. 2014, Kujirai et al. 2018, Johnson et al. 2025c). Resolving whether these candidates function as redox sensors will require further experimental characterization. Structural prediction tools can identify redox-sensitive intrinsically disordered regions and assess whether modified cysteines occupy positions likely to affect DNA binding (Erdős et al. 2019, Pis Diez et al. 2022, Hekkelman et al. 2023). Pairing these predictions with high-throughput sequencing links protein modifications to downstream regulons (Riediger et al. 2019, Kariyazono and Osanai 2022, Johnson et al. 2025c). Structural proteomics approaches, including limited proteolysis-mass spectrometry, thermal proteome profiling, and redox proteomics, extend this toolkit to proteome-wide discovery of conformationally dynamic and redox-sensitive proteins, identifying regulatory states invisible to abundance-based methods alone (Sarkar et al. 2025). Together, these approaches enable systematic identification of redox-responsive regulatory circuits, moving the field beyond the case-by-case characterization that has defined it to date.

These same approaches will be needed to convert native redox sensors into engineered parts. In heterotrophic hosts, redox-sensing transcription factors such as the [2Fe-2S]-containing SoxR and the NADH-responsive Rex have been repurposed as biosensors and feedback circuits that autonomously adjust pathway expression in response to metabolic state (Chou and Keasling 2013, Dahl et al. 2013, Siedler et al. 2014). Quantitative dose-response and promoter architecture data from systematic characterization would enable similar engineering of regulators such as SufR and RpaB in cyanobacteria. The photosynthetic electron transport chain provides the most direct biological link between light energy and reductive biosynthesis; transcriptional circuits that monitor electron flux through this chain would enable autonomous, light-responsive production control with no equivalent in heterotrophic systems.
